# Transforming Aquaculture through Vaccination: A Review on Recent Developments and Milestones

**DOI:** 10.3390/vaccines12070732

**Published:** 2024-07-01

**Authors:** Iosif Tammas, Konstantina Bitchava, Athanasios I. Gelasakis

**Affiliations:** 1 Laboratory of Applied Hydrobiology, Department of Animal Science, Agricultural University of Athens, 11855 Athens, Greece; stud217095@aua.gr; 2 Laboratory of Anatomy & Physiology of Farm Animals, Department of Animal Science, Agricultural University of Athens, 11855 Athens, Greece

**Keywords:** aquaculture, vaccines, vaccinology, vaccination, adjuvants, sustainability, pathogens, immunization

## Abstract

Aquaculture has rapidly emerged as one of the fastest growing industries, expanding both on global and on national fronts. With the ever-increasing demand for proteins with a high biological value, the aquaculture industry has established itself as one of the most efficient forms of animal production, proving to be a vital component of global food production by supplying nearly half of aquatic food products intended for human consumption. As in classic animal production, the prevention of diseases constitutes an enduring challenge associated with severe economic and environmental repercussions. Nevertheless, remarkable strides in the development of aquaculture vaccines have been recently witnessed, offering sustainable solutions to persistent health-related issues challenging resilient aquaculture production. These advancements are characterized by breakthroughs in increased species-specific precision, improved vaccine-delivery systems, and innovations in vaccine development, following the recent advent of nanotechnology, biotechnology, and artificial intelligence in the -omics era. The objective of this paper was to assess recent developments and milestones revolving around aquaculture vaccinology and provide an updated overview of strengths, weaknesses, opportunities, and threats of the sector, by incorporating and comparatively discussing various diffuse advances that span across a wide range of topics, including emerging vaccine technologies, innovative delivery methods, insights on novel adjuvants, and parasite vaccine development for the aquaculture sector.

## 1. Introduction

The recent advent of aquaculture has marked a profound paradigm shift in the global approach to aquatic food production during the last decades, despite the practice stemming from ancient Chinese tradition going back thousands of years [1]. In response to the ever-increasing demands for proteins with a high biological value and the ecological pressure exerted by fisheries, aquaculture has emerged as a vital solution for global nutrition security, signifying an important transformation to the way the world meets its needs [2,3]. As per the Food and Agriculture Organization of the United Nations (FAO), this practice involves the farming of aquatic organisms, including fish, mollusks, crustaceans, and aquatic plants, under meticulously protected and managed environments. According to the latest reports, aquaculture produces more than half of aquatic food products intended for human consumption [4]. With the potential to alleviate overfishing, promote food security, and provide economic opportunities, the aquaculture industry stands as a pivotal milestone to the enduring pursuit for sustainable aquatic food production in the modern world [5,6].

The rise of intensive aquaculture has been heavily linked with the use of antibiotics as a means of battling detrimental disease outbreaks that have sparked due to increased rearing densities [7,8]. However, many concerns have been raised about their adverse effects not only on humans but also on environmental health. Indeed, under the doctrine of ‘One-Health’, where human, animal, and environmental health are closely interconnected, aquaculture specialists have been actively searching for alternatives to boost the sustainability of the aquaculture industry by focusing more on disease prevention and less on the use of antimicrobials [9,10,11]. In this quest for sustainable disease management, vaccination rises as the leading solution, since it is the most efficient tool for disease prevention in humanity’s arsenal up to date.

Modern vaccination traces its roots to the pioneering work of Edward Jenner, yet the practice in aquaculture is a considerably more recent development, beginning in 1938 with Snieszko’s study on carp immunization and continuing with Duff’s 1942 report marking the first written publication in the English language [12,13,14]. In 1976, the first commercial vaccine for aquaculture was licensed in the U.S., and today, more than 30 vaccines are available for use in the global market, according to the available literature estimates [15,16,17,18]. Advances in scientific and technological expertise, nevertheless, have accelerated the development process of aquaculture vaccines in recent years, leading to an increasing number of available options for the protection of aquatic organisms against pathogens, marking a significant milestone in aquaculture vaccinology.

As an end goal, this paper aims to provide an extensive and comprehensive review on the recent advances made in the field of aquaculture vaccines. By collectively bringing together many disparate advances that span across different aspects of vaccinology in a single and up-to-date article, the objective is to provide the reader with a unified perspective that will foster a better understanding on the current state of aquaculture vaccinology. Ultimately, this understanding will not only aid in illuminating emerging trends in the industry but also in evaluating implications for future advancements, challenges, and directions. Transitioning from the basis of vaccination, however, to exploring the intricacies of the immune system, understanding how aquatic organisms defend themselves against pathogen threats becomes paramount in understanding how vaccines work.

## 2. An Overview of Aquatic Organism Immunology

### 2.1. Structural and Functional Insights into the Teleost Immune System

Fish form an integral link between vertebrates and invertebrates when it boils down to the phylogenetic spectrum of evolution [19]. Despite the term not having any official entity in today’s systematic biology, fish include over 40,000 species belonging to various taxonomic groups and homotaxa. Under this scope, many differences are presented not only in terms of anatomy but also in terms of the structure and physiological function of the immune system [20]. This diversity makes fish an excellent model for comparative immunology, helping elucidate many important aspects and details of the evolution of the immune system among different species [21,22].

#### 2.1.1. Primary and Secondary Immune Organs in Teleosts

Aquaculture is predominantly based on the farming of teleost fish, which forms the most populous infraclass of the ray-finned fishes (class *Actinopterygii*) [23]. The immune system of teleost fish comprises two primary organs, the T-cell-producing thymus and the B-cell-producing head kidney. Apart from these two organs, the function of the teleost immune system is further supported by secondary organs, such as the spleen and the mucosal-associated lymphoid tissues (MALTs), located in the skin (SALT), the gills (GIALT), the gut (GALT), and the nasopharynx (NALT). These tissues contain diffuse immune cells, each containing distinct and unique phenotypes. This variation in phenotype is contingent upon the type of tissue, particularly because of the tropism and the specificity of the pathogens encountered at their respective locations [24,25,26,27]. Unlike mammals, teleost fish do not have any bone marrow, nor do they display any organized lymphoid structures like Peyer’s patches [28]; however, the existence of intestinal epithelial cells (IELs) with explicit immune function has been suggested, with recent studies highlighting the existence of additional lymphoid tissues located in the stomach, the gallbladder, the swim bladder, and the ocular mucosa [29,30].

In terms of function, the teleost immune system comprises two essential mechanisms, the innate and the adaptive immunity, following essentially a similar motif to that observed in higher vertebrates. Innate immunity comprises the first line of defense against pathogen infections, faring against them in a non-specific manner, whereas the adaptive immunity picks up against persisting infections in a highly specialized response to neutralize the impeding pathogen threat [31,32].

#### 2.1.2. The Teleost Innate Immune System

**Figure 1:**
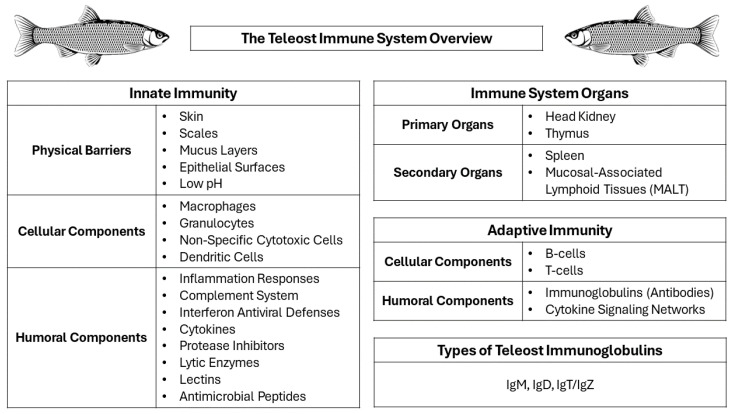
A schematic illustration summarizing the main components of the teleost immune system, including anatomical, innate, and adaptive immune elements.

The innate immune system is vital to teleosts and comprises three main components: physical barriers (skin, scales, mucus layers, gills, and epithelial linings); cellular factors (macrophages, granulocytes, non-specific cytotoxic cells, and dendritic cells); and humoral factors (various signaling and defensive macromolecules) [33,34,35,36,37,38]. These elements orchestrate the function of immune responses against pathogens, which begins with the recognition of pathogen-associated molecular patterns (PAMPs) through specific immune receptors [39,40]. Despite being an evolutionary older mechanism than adaptive immunity, the innate arm of the immune system is essential to teleost fish, due to their poikilothermic physiology and the microbe-rich aquatic environment [41]. By working in harmonious conjunction with the adaptive arm, innate immunity provides an immediate reaction to impeding pathogen threats until adaptive immune responses can activate and deal with the threat in a specific manner [20,39]. A schematic overview illustrating all elements of the teleost immune system can be found in Figure 1.

#### 2.1.3. Adaptive Immunity in Teleosts

Once innate immune responses are no longer able to restrict the pathogen threat, the adaptive immune system is activated in teleosts, producing highly specialized antibodies and preserving immune memory through specific memory cells in a phenomenon known collectively as immune memory [42]. The key cellular components of adaptive immunity in teleosts consist of B cells, responsible for antibody production and immune memory, and T cells, which facilitate both functional and regulatory roles. The humoral components include the secreted antibodies, known as immunoglobulins, where three types have been highlighted in teleosts according to the literature [27,28,29]. Pathogen binding to B-cell receptors (BCRs) initiates B-cell differentiation into plasma and memory B cells, aided by T cells also binding to pathogens through their own T-cell receptors (TCRs) during the phenomenon of antigen presentation [33,42]. Antigen-presenting cells (APCs), like macrophages, B cells, and dendritic cells (DCs), bind to antigens via their surface-expressed MHC class II proteins and display them to T cells, leading to their differentiation to CD4+ Th cells, which facilitate antibody production and antigen-specific humoral responses through intricate signaling cascades [22,23,32,43]. Similarly, teleost cellular adaptive immune responses materialize through the binding of pathogens by APCs through MHC class I proteins, leading to the differentiation of T cells into CD8+ cytotoxic Tc cells, which facilitate cell apoptosis and memory T-cell generation for long-term protection [23,32,44,45,46,47].

Overall, vaccination aims to stimulate the teleost fish immune system and leads to the production of specialized antibodies and immune memory cells against the pathogen of interest, conferring long-term protection against infections by leveraging off the capabilities of the adaptive immune system to elicit protection against diseases through a tailored defense network that recognizes subsequent and reoccurring encounters with aquatic pathogens. As such, an effective numerical value for efficient immunization in teleosts can be evaluated through the detection of antigen-specific antibody titers in vaccinated organisms, though investigating the effects of vaccines in the context of innate immune responses can be of value as well, helping to elucidate the intricate mechanisms behind fish immunization.

### 2.2. The Peculiar Case of Aquatic Invertebrates

Bivalve mollusks and decapod crustaceans, the primary aquatic invertebrates in aquaculture, rely on an immune system that is simpler in design when compared to that of fish, since it encompasses only an innate function that comprises physical barriers (shells and chitin exoskeletons) and cellular and humoral components [48,49,50,51]. Hemocytes, found in hemolymphs, play a crucial role in immune functions and facilitate a plethora of processes such as phagocytosis, cell lysis, encapsulation, apoptosis, autophagy, and shell healing via mineralization [52,53,54,55,56,57]. Decapod crustacean hemocytes include hyaline, granular, and semi-granular cells, whereas bivalve mollusks only exhibit granular and agranular cells [49,52,56,57]. Pathogen recognition by specific pattern recognition receptors (PRRs) triggers immune responses, involving hemocytes, and the secretion of various defense molecules, like antimicrobial peptides (AMPs) [48,51,58,59,60,61,62]. The prophenoloxidase (ProPO) system leads to melanin accumulation and cytotoxic molecule secretion, while the clotting system supports hemolymph circulation and collaborates with the process of melanization and the action of AMPs [53,54,61,63,64]. It is evident that hemocytes stand as crucial elements for aquatic invertebrate immunity, yet hemopoiesis in these organisms still constitutes a research area under investigation. Recent studies suggest that the gills are crucial for bivalve hemopoiesis [57,65], whereas unique hemopoietic tissues exist in specific regions of decapod crustaceans [66].

Albeit supported by a primarily innate immune system, aquatic invertebrates exhibit immune memory-like characteristics, leading to the induction of quasi-specific immune responses against pathogens, according to recent literature insights [48,67,68,69]. As such, aquatic invertebrates can indeed be primed, essentially opening the gateways to immunization within their realm as well. Although not as extensively researched and applied, aquatic invertebrate priming includes various approaches, including immunostimulatory or immunomodulatory feed manipulations, RNA interference technology, and even direct immunization, as summarized by [48,69,70,71]. However, logistical aspects of this endeavor remain unclear, as their effectiveness and cost-related equilibrium need to be further elucidated for their industrial application to take effect.

## 3. The Current Landscape of Commercially Available Aquaculture Vaccines

### 3.1. Conventional and Commercially Available Aquaculture Vaccine Technologies

As far as aquatic animal health is concerned, the majority of conventional and commercially available vaccines in aquaculture are still based on Pasteurian principles, where a pathogen is isolated, killed, or inactivated and then formulated into a vaccine ready for administration via various routes. With the recent scientific and technological bloom of the 21st century, however, progressively more vaccine technologies have emerged, with many of them being implemented as platforms for the development of efficacious vaccines in the aquaculture industry as well [72]. Currently, there are four main types of vaccine technologies used for the development of commercial aquaculture vaccines: whole-cell inactivated vaccines, live-attenuated vaccines, subunit vaccines, and DNA vaccines. Below, the respective available types of commercially available aquaculture vaccine technologies are discussed, and a collective table containing major vaccines in the global aquaculture market is provided in Table 1.

#### 3.1.1. Whole-Cell Inactivated Vaccines

Whole-cell inactivated vaccines constitute the most common vaccine type in the aquaculture industry [15,73]. These vaccines contain live pathogens that are cultured and inactivated by using chemical or physical means, such as heat, radiation, and more commonly, formalin. This process nullifies the virulence of the microorganisms, while simultaneously retaining the antigenic properties that are responsible for eliciting an immune response to the host [72]. As a result, whole-cell inactivated vaccines offer increased safety profiles, though their immunogenicity is typically considered to be rather low. For this reason, the use of auxiliary substances called adjuvants, or several booster shots, are frequently required to elicit sufficient levels of immunization upon administration [15,72].

The effectiveness of these vaccines is further compromised, especially when used against pathogens that are heterogenous in nature [16,74]. As such, it is important to consider both the host’s and the pathogen’s species when developing a novel whole-cell inactivated vaccine. In addition, this vaccine technology tends to fall short against intracellular and viral pathogens, meaning that it is typically restricted to a certain range of bacterial pathogens overall [15,74]. As a significant counterbalance, however, it is important to note that these vaccines offer an affordable and quite straightforward platform for vaccine development and production. Whole-cell inactivated vaccines boast enhanced stability during both storage and transportation within the cold-chain process [15]. This poses a significant advantage for this type of vaccine technology that has contributed immensely to their widespread adoption and utilization within the aquaculture industry.

The mitigation of logistical challenges and cost-related issues are important aspects to a vaccine’s reliability, especially within the dynamic context of aquatic health management. Consequently, whole-cell inactivated vaccines have rightfully earned their position as the golden standard in aquaculture vaccination worldwide, despite their seeming limitations. Today, this technology offers commercially available solutions to a wide range of important aquatic diseases, including enteric red mouth (ERM) disease, furunculosis, pasteurellosis, lactococcosis, and vibriosis.

#### 3.1.2. Live-Attenuated Vaccines

The second type of vaccine technology used for the production of commercially available vaccines in aquaculture comprises live-attenuated vaccines. This pivotal category incorporates vaccines that contain viable pathogens that retain their replication capabilities but have undergone modifications to significantly decrease their pathogenic capacity. The attenuation of these pathogens can be carried out in various ways, such as the use of chemical or physical means (chemical or physical attenuation), serial passaging through heterologous systems, or the utilization of more modern biotechnological approaches, such as genetic engineering and biotechnological manipulation [73].

Contrary to whole-cell inactivated vaccines, live-attenuated vaccines do not lose their ability to replicate once inside the host. This enables them to mimic infections that occur naturally in the hosts and induce potent immune responses that can activate both cellular and humoral aspects of immunity. As a result, the main advantage of these vaccines boils down to their increased immunogenicity and their ability to provide long-lasting immunization, effectively minimizing the need for additional adjuvants and several booster shots to confer adequate protection [15,72,75]. Despite their promising potential, however, live-attenuated vaccines do not come without their disadvantages.

Since they contain viable pathogens, the administration of these vaccines is often contraindicated in immunocompromised organisms. In addition, there are rare, yet significant, risks associated with the potential of attenuated strains reverting to virulent forms [15,76]. Hence, the meticulous selection, characterization, and attenuation of pathogen strains is imperative to address all pertinent parameters during the development of a novel live-attenuated vaccine. Logistically speaking, the development of live-attenuated vaccines far surpasses the demands of whole-cell inactivated vaccines, since it requires more intricate technology and stringent cold-chain storing management. This suggests that despite being effective, live-attenuated vaccines require careful assessment of their associated risks and logistical challenges, underscoring the importance of continual scientific exploration for alternative and novel vaccine technologies to be implemented in the future of aquaculture vaccinology.

#### 3.1.3. Subunit Vaccines

Subunit vaccines represent a cutting-edge paradigm in modern vaccinology, forming a significant milestone of the recent scientific and technological bloom of the 21st century. Characterized by their high specificity, these vaccines do not contain any whole pathogens. Instead, subunit vaccines utilize inherently immunogenic purified fragments from microorganisms that can elicit immune responses to hosts upon administration. This meticulous selection essentially nullifies the risk of using a viable inactivated pathogen, therefore enhancing the safety profile of this vaccine technology in comparison to live-attenuated vaccines [15,72,76].

The inherent precision of these vaccines allows for the customization of the immunogenic pathogen compartments, optimizing the elicitation of the desired immune response while simultaneously minimizing the associated risks. Additionally, subunit vaccines can circumvent the previous restrictions inherent in administering live-attenuated vaccines to immunocompromised organisms [72,76]. The ongoing advancements in the fields of biotechnology and molecular biology have accelerated the development and refinement of subunit vaccines in recent times, often positioning them as ideal vaccine candidates du-ring novel vaccine development in veterinary sciences, especially in situations where the culturing of pathogens proves difficult [76].

A common approach to produce a subunit vaccine currently involves the expression of recombinant antigens through the utilization of genetically modified organisms or cell line cultures [15,72,76]. This process can prove quite resource and labor intensive, especially in regions that are financially challenged. The difficulties of the widespread adoption of subunit vaccines in the aquaculture industry become even more pronounced, especially when considering the predominant economic conditions of the regions where aquaculture production plays a pivotal role. In addition, because of the intrinsic nature of their design, subunit vaccines are often not able to induce robust immune responses, because they only contain specific and isolated fragments from whole pathogens that are unable to emulate the complex antigenic effects of live pathogens [15].

In a paradoxical manner, the high specificity of this vaccine technology comes at a cost and poses as a constraint on its overall efficacy, ultimately leading to the inevitable necessity of adjuvants and several booster shots to achieve adequate levels of protection against diseases. Consequently, striking a balance between cost effectiveness and complete levels of protection remains a challenge in the development and implementation of subunit vaccines, especially in the context of aquaculture. Despite these difficulties, however, there are several commercially available subunit vaccines that are efficient in preventing aquatic diseases, particularly those of viral etiological agents.

#### 3.1.4. DNA Vaccines

The most recent addition to the arsenal of commercially available aquaculture vaccines is represented by nucleic acid vaccines and, in particular, DNA vaccines. These vaccines do not contain any microorganisms, nor do they contain antigenic fragments. Instead of relying on traditional methods, DNA vaccines encompass the introduction of genetic material that encodes specific pathogen antigens directly inside the host. This genetic payload can confer protection by stimulating the host’s cells to express the targeted antigens of interest, therefore prompting a robust immune response in return [15,72,74].

The antigen genes are usually encoded into expression vectors, most often plasmids, alongside all the genetic elements necessary for the initiation, regulation, and termination of gene expression inside eukaryotic cells [15,72]. Following administration, the selected genes are subsequently transcribed and then translated into the desired antigens by adhering to the principles that dictate the central dogma of molecular biology. In sequence, these antigens are recognized by the host’s immune system and elicit strong immune responses, activating both cellular and humoral immunity. This response is very potent in general and can confer robust and long-lasting protection in teleost fish [15,72,74].

The use of DNA vaccine technology offers many advantages, being considered overall safe for administration, rapid in production, and suitable for tailoring vaccines to combat a wide spectrum of aquatic pathogens, especially intracellular ones [77]. However, it is important to note that the mechanisms behind the potential integration of foreign DNA in the host’s genome are unclear so far in the context of aquatic organisms [78]. Due to its relative novelty, this vaccine technology is challenged with both consumer acceptance and regulatory and legal scrutiny that impede the widespread implementation of DNA vaccines in the aquaculture industry [77]. As researchers strive to refine this vaccine technology, it is evident that DNA vaccines hold promise for enhancing disease control and resistance within aquaculture settings, thus contributing to the sustainable and healthy cultivation of aquatic species in the modern era. In Canada, a DNA vaccine against the infectious hematopoietic necrosis virus (IHNV) is commercially available, whereas recently, in 2017, a DNA vaccine against the Salmonid alphavirus SAV-3 was licensed in Europe, marking a significant milestone for global aquaculture vaccinology [15,16,72,74,77].

**Table 1:** A collective overview of major and currently commercially available vaccines in the global aquaculture market. To our knowledge, this is the first collective attempt to pinpoint licensed vaccines in the current literature, showcasing a significant milestone for the industry and an increase from previous literature estimates.

Vaccine	Disease	Antigen	Technology	Delivery	Species	Region
**PHARMAQ (https://pharmaq.com/en/pharmaq/our-products) (accessed on 20 June 2024)**						
ALPHA DIP ^®^ 2000	Pasteurellosis Vibriosis	Inactivated cultures of *Listonella (Vibrio) anguillarum* (serotype O1) and *Photobacterium damselae* subsp. *piscicida*	Inactivated	Immersion	Sea Bass	Europe
ALPHA DIP ^®^ Vib	Vibriosis	Inactivated bacteria culture of *Listonella (Vibrio) anguillarum* (serotype O1)	Inactivated	Immersion	Sea Bass	Europe
ALPHA DIP ^®^ Vibrio	Vibriosis	Inactivated bacteria culture of *Listonella (Vibrio) anguillarum* (serotype O1)	Inactivated	Immersion	Sea Bass	West Asia
Alpha ERM Salar	Enteric Red Mouth	Inactivated culture of *Yersinia ruckeri* (serotype O1b)	Inactivated	Injection	Atlantic Salmon	Europe
Alpha ERM Salar (Dip)	Enteric Red Mouth	Inactivated culture of *Yersinia ruckeri* (serotype O1b)	Inactivated	Immersion	Atlantic Salmon	Europe N. America
ALPHA JECT LiVac ^®^ SRS	Salmon Rickettsial Syndrome	Live culture of *Piscirickettsia salmonis* (strain AL20542)	Live Attenuated	Injection	Salmonids	S. America
ALPHA JECT micro ^®^ 1 ISA	Infectious Salmon Anemia	Inactivated Infectious Salmon Anemia Virus (strain ALV301)	Inactivated	Injection	Atlantic Salmon	S. America
ALPHA JECT micro ^®^ 1 Noda	Viral Nervous Necrosis	Inactivated Red Grouper Necrosis Virus (strain ALV1107)	Inactivated	Injection	Sea Bass	Europe
ALPHA JECT micro ^®^ 1 PD	Pancreas Disease	Inactivated Salmon Pancreas Disease Virus (strain ALV405)	Inactivated	Injection	Atlantic Salmon	Europe
ALPHA JECT micro ^®^ 1 Tila	Streptococcosis	Inactivated bacteria culture of *Streptococcus agalactiae*	Inactivated	Injection	Tilapia	Asia S. America
ALPHA JECT micro ^®^ 2	Infectious Pancreatic Necrosis Salmon Rickettsial Syndrome	Inactivated cultures of Infectious Pancreatic Necrosis Virus (strain ALV013) and *Piscirickettsia salmonis* (strain AL10015)	Inactivated	Injection	Salmonids	S. America
ALPHA JECT micro ^®^ 2000	Pasteurellosis Vibriosis	Inactivated cultures of *Listonella (Vibrio) anguillarum* (serotype O1) and *Photobacterium damselae* subsp. *piscicida*	Inactivated	Injection	Sea Bass	Europe West Asia
ALPHA JECT micro ^®^ 3	Infectious Pancreatic Necrosis Salmon Rickettsial Syndrome Vibriosis	Inactivated cultures of Infectious Pancreatic Necrosis Virus (strain ALV103), *Piscirickettsia salmonis* (strain AL10015), and *Vibrio ordalii* (strain AL510)	Inactivated	Injection	Atlantic Salmon	S. America
ALPHA JECT micro ^®^ 4	Coldwater Vibriosis Furunculosis Vibriosis	Inactivated bacteria cultures of *Aeromonas salmonicida* subsp. *salmonicida*, *Listonella (Vibrio) anguillarum* (serotypes O1 and O2), and *Vibrio salmonicida*	Inactivated	Injection	Atlantic Salmon	N. America
ALPHA JECT micro ^®^ 4-2	Furunculosis Infectious Pancreatic Necrosis Infectious Salmon Anemia Vibriosis	Inactivated cultures of *Aeromonas salmonicida* subsp. *Salmonicida* (strain AL2017), Infectious Pancreatic Necrosis Virus (strain ALV103), and *Vibrio ordalii* (strain AL510)	Inactivated	Injection	Atlantic Salmon	S. America
ALPHA JECT micro ^®^ 5	Coldwater Vibriosis Furunculosis Vibriosis Winter Sore	Inactivated cultures of *Aeromonas salmonicida* subsp. *salmonicida*, *Listonella (Vibrio) anguillarum* (serotypes O1 and O2a), *Vibrio salmonicida*, and *Moritella viscosa*	Inactivated	Injection	Atlantic Salmon	Europe N. America
ALPHA JECT micro ^®^ 6	Coldwater Vibriosis Furunculosis Infectious Pancreatic Necrosis Winter Sore	Inactivated cultures of *Aeromonas salmonicida* subsp. *salmonicida*, *Listonella (Vibrio) anguillarum* (serotypes O1 and O2a), *Vibrio salmonicida*, *Moritella viscosa* and Infectious Pancreatic Necrosis Virus (serotype sp.)	Inactivated	Injection	Atlantic Salmon	Europe
ALPHA JECT micro ^®^ 7 ILA	Coldwater Vibriosis Furunculosis Infectious Pancreatic Necrosis Infectious Salmon Anemia Vibriosis Winter Sore	Inactivated cultures of *Aeromonas salmonicida* subsp. *salmonicida*, *Listonella (Vibrio) anguillarum* (serotypes O1 and O2a), *Vibrio salmonicida*, *Moritella viscosa*, Infectious Pancreatic Necrosis Virus (serotype sp.) and Infectious Salmon Anemia Virus	Inactivated	Injection	Atlantic Salmon	Europe
ALPHA JECT micro ^®^ 7 ISA	Coldwater Vibriosis Furunculosis Infectious Pancreatic Necrosis Infectious Salmon Anemia Vibriosis Winter Sore	Inactivated cultures of *Aeromonas salmonicida* subsp. *salmonicida*, *Listonella (Vibrio) anguillarum* (serotypes O1 and O2a), *Vibrio salmonicida*, *Moritella viscosa*, Infectious Pancreatic Necrosis Virus (*serotype* sp.) and Infectious Salmon Anemia Virus	Inactivated	Injection	Atlantic Salmon	N. America
ALPHA JECT ^®^ 3000	Furunculosis Vibriosis	Inactivated bacteria cultures of *Aeromonas salmonicida* subsp. *Salmonicida* (strain AL2017) and *Listonella (Vibrio) anguillarum* (Serotypes O1 and O2a)	Inactivated	Injection	Salmonids	Europe
ALPHA JECT ^®^ 1000	Infectious Pancreatic Necrosis	Inactivated culture of Infectious Pancreatic Necrosis Virus (strain ALV103)	Inactivated	Injection	Salmonids	S. America
ALPHA JECT ^®^ 2000	Pasteurellosis Vibriosis	Inactivated cultures of *Listonella anguillarum* (serotype O1) and *Photobacterium damselae* subsp. *piscicida*	Inactivated	Injection	Sea Bass	Europe West Asia
ALPHA JECT ^®^ 3 FPV	Furunculosis Pasteurellosis Vibriosis	Inactivated cultures of *Listonella (Vibrio) anguillarum* (serotype O1), *Photobacterium damselae* subsp. *piscicida* (strain AL5051), and *Aeromonas salmonicida* subsp. *Salmonicida* (strain AL2017)	Inactivated	Injection	Sea Bass	Europe
ALPHA JECT ^®^ 5-1	Furunculosis Infectious Pancreatic Necrosis Infectious Salmon Anemia Salmon Rickettsial Syndrome Vibriosis	Inactivated cultures of Infectious Samon Anemia Virus (strain ALV301), Infectious Pancreatic Necrosis Virus (strain ALV103), *Piscirickettsia salmonis* (strain AL10005), *Aeromonas salmonicida* subsp. *Salmonicida* (strain AL2017), and *Vibrio ordalii* (strain AL510)	Inactivated	Injection	Atlantic Salmon	S. America
ALPHA JECT ^®^ 6-2	Coldwater Vibriosis Furunculosis Infectious Pancreatic Necrosis Vibriosis Winter Sore	Inactivated cultures of *Aeromonas salmonicida* subsp. *Salmonicida*, *Vibrio salmonicida*, *Listonella (Vibrio) anguillarum* (serotype O1), and *Moritella viscosa* Infectious Pancreatic Necrosis Virus (serotype sp.)	Inactivated	Injection	Atlantic Salmon	Europe
ALPHA JECT ^®^ IPNV-Flavo 0.025	Flavobacteriosis Infectious Pancreatic Necrosis	Inactivated cultures of *Flavobacterium psychrophilum* (strain AL20055) and Infectious Pancreatic Necrosis Virus (*serotype* sp.)	Inactivated	Injection	Atlantic Salmon	S. America
ALPHA JECT ^®^ Moritella	Winter Sore	Inactivated bacteria culture of *Moritella viscosa* (strain AL21355)	Inactivated	Injection	Atlantic Salmon	Europe
ALPHA JECT ^®^ Panga 2	Enteric Septicemia Disease Motile Aeromonas Septicemia	Inactivated cultures of *Edwardsiella ictaluri* (strain AL20658) and *Aeromonas hydrophila* (Serotypes A and B)	Inactivated	Injection	Pangasius	Asia
**MERCK-MSD (https://www.msd-animal-health.com/products/aquaculture/aquaculture-products/) (accessed on 20 June 2024)**						
AQUAVAC ^®^ ERM	Enteric Red Mouth	Inactivated culture of *Yersinia ruckeri* (Hagerman type 1 strain)	Inactivated	Injection Oral Immersion	Trout	Europe
AQUAVAC ^®^ YER	Yersiniosis	Inactivated culture of *Yersinia ruckeri* (Hagerman type 1 strain)	Inactivated	Injection	Atlantic Salmon	Europe
AQUAVAC ^®^ Vibrio Pasteurella	Pasteurellosis Vibriosis	Inactivated cultures of *Listonella (Vibrio) anguillarum* (Serotypes O1 and O2) and *Photobacterium damselae* subsp. *piscicida*	Inactivated	Injection	Sea Bass	Europe
AQUAVAC ^®^ Vibrio	Vibriosis	Inactivated culture of *Listonella (Vibrio) anguillarum* (serotype O1) and *Vibrio ordalii* (strain MSC275)	Inactivated	Injection Immersion Oral	Trout Sea Bass	Europe
AQUAVAC ^®^ ESC	Enteric Septicemia	Live avirulent culture of *Edwardsiella ictaluri* (strain RE-33)	Live Attenuated	Immersion	Catfish	N. America
AQUAVAC ^®^ IridoV	Iridoviral Infection Mortality	Inactivated iridovirus	Inactivated	Injection	Tilapia Sea Bass	Asia
AQUAVAC ^®^ Strep Sa	Streptococcosis	Inactivated culture of *Streptococcus agalctiae* (strain TI513)	Inactivated	Injection	Tilapia	Asia S. America
AQUAVAC ^®^ Step Sa1	Streptococcosis	Inactivated culture of *Streptococcus agalctiae* (Serotypes Ia and III)	Inactivated	Injection	Tilapia	Asia S. America
AQUAVAC ^®^ Step Si	Streptococcosis	Inactivated culture of *Streptococcus iniae* (strain SB430)	Inactivated	Injection	Tilapia Sea Bass	Asia S. America
AQUAVAC ^®^ RELERA	Enteric Red Mouth	Inactivated cultures of *Yersinia ruckeri* (Hagerman type 1 and EX5 biotype strains)	Inactivated	Injection Immersion	Trout	Europe
AQUAVAC ^®^ PD3	Furunculosis Infectious Pancreatic Necrosis Salmon Pancreas Disease	Inactivated cultures of *Aeromonas salmonicida*, Infectious Pancreatic Virus (*serotype* sp.), and Salmon Pancreas Disease Virus (strain F93-125)	Inactivated	Injection	Atlantic Salmon	Europe
AQUAVAC ^®^ PD7	Furunculosis Infectious Pancreatic Necrosis Salmon Pancreas Disease Vibriosis Wound Disease	Inactivated cultures of *Aeromonas salmonicida*, Infectious Pancreatic Virus (serotype Sp), Salmon Pancreas Disease Virus (strain F93-125), *Listonella (Vibrio) anguillarum* (Serotypes O1 and O2a), *Aliivibrio salmonicida*, and *Moritella viscosa*	Inactivated	Injection	Atlantic Salmon	Europe
AQUAVAC ^®^ SARISTIN 2	Infectious Pancreatic Necrosis Salmon Rickettsial Syndrome	Recombinant Infectious Pancreatic Necrosis Virus VP2 protein and recombinant *Piscirickettsia salmonis* lipoprotein	Subunit	Injection	Salmonids	S. America
AQUAVAC ^®^ 6	Furunculosis Infectious Pancreatic Necrosis Vibriosis Wound Disease	Inactivated cultures of Infectious Pancreatic Virus (*serotype* sp.) *Aeromonas salmonicida* subsp. *Salmonicida*, *Vibrio* salmonicida, *Listonella (Vibrio) anguillarum* (serotype O1 and O2a), and *Moritella viscosa*	Inactivated	Injection	Atlantic Salmon	Europe
AQUAVAC ^®^ IPN Oral	Infectious Pancreatic Necrosis	Recombinant Infectious Pancreatic Necrosis Virus Proteins VP2 and VP3	Subunit	Oral	Atlantic Salmon	S. America
AQUAVAC ^®^ Photobac Prime	Pasteurellosis	Inactivated culture of *Photobacterium damselae* subsp. *piscicida*	Inactivated	Immersion	Sea Bass Sea Bream	Europe
**ELANCO (https://www.elancoaquaglobal.com/us/en) (accessed on 20 June 2024)**						
Clynav^TM^	Pancreas Disease	Recombinant plasmid encoding Salmon Pancreas Disease Virus Proteins	DNA	Injection	Atlantic Salmon	Europe
Pentium Forte Plus^TM^	Coldwater Vibriosis Furunculosis Infectious Pancreatic Necrosis Winter Sore Vibriosis	Inactivated cultures of Infectious Pancreatic Virus, *Aeromonas salmonicida* subsp. *Salmonicida*, *Listonella (Vibrio) anguillarum* (serotype O1 and O2a), and *Moritella viscosa*	Inactivated	Injection	Atlantic Salmon	Europe
Apex-IHN ^®^	Infectious Hematopoietic Necrosis	Recombinant plasmid encoding Infectious Hematopoietic Necrosis Virus Proteins	DNA	Injection	Salmonids	N. America
FORTE ^®^ Micro	Cold Water Vibriosis Furunculosis Vibriosis	Inactivated cultures of *Aeromonas salmonicida*, *Listonella (Vibrio) anguillarum* (Serotypes 1 and 2), *Vibrio ordalii*, and *Vibrio salmonicida* (Serotypes 1 and 2)	Inactivated	Injection	Salmonids	N. America
Forte ^®^ VII	Coldwater Vibriosis Furunculosis Infectious Salmon Anemia Vibriosis	Inactivated cultures of Infectious Salmon Anemia Virus, *Aeromonas salmonicida*, *Listonella (Vibrio) anguillarum* (Serotypes 1 and 2), *Vibrio ordalii*, and *Vibrio salmonicida* (Serotypes 1 and 2)	Inactivated	Injection	Salmonids	N. America
Renogen ^®^	Bacterial Kidney Disease	Lyophilized live culture of a microorganism that shares common antigenic determinants with *Renibacterium salmonarum*	Live Attenuated	Immersion	Salmonids	N. America S. America
Birnagen Forte ^®^ 2	Infectious Pancreatic Necrosis Salmon Rickettsial Syndrome	Inactivated culture of Infectious Pancreatic Necrosis Virus and recombinant *Piscirickettsia salmonis* HSP70, HP60, and FLG G2 proteins	Inactivated Subunit	Injection	Salmonids	S. America
Pentium Forte Plus ILA ^®^	Furunculosis Infectious Pancreatic Necrosis Infectious Salmon Anemia Vibriosis	Inactivated cultures of *Aeromonas salmonicida*, *Listonella (Vibrio) anguillarum* (Serotypes O1 and O2), Infectious Pancreatic Necrosis Virus, and Infectious Salmon Anemia Virus	Inactivated	Injection	Atlantic Salmon	S. America
**HIPRA (https://www.hipra.com/en/animal-health) (accessed on 20 June 2024)**						
Icthiovac ^®^ VNN	Viral Nervous Necrosis	Inactivated *Betanodavirus* (strain 1103)	Inactivated	Injection	Sea Bass	Europe
Icthiovac ^®^ PD	Pasteurellosis	Inactivated culture of *Photobacterium damselae* subsp. *Piscicida* (strains DI-21 and lt-1)	Inactivated	Immersion	Sea Bream	Europe
Icthiovac ^®^ LG	Lactococcosis	Inactivated culture of *Lactococcus garvieae* (strain TW446.B3)	Inactivated	Injection	Trout	Europe
Icthiovac ^®^ VR/PD	Pasteurellosis Vibriosis	Inactivated cultures of *Listonella (Vibrio) anguillarum* (Serotypes O1, O2a, and O2b) and *Photobacterium damselae* subsp. *piscicida* (strain DI21)	Inactivated	Injection	Sea Bass	Europe
Icthiovac ^®^ TM	Tenacibaculosis	Inactivated *Tenacibaculum maritimum* (strain LPV 1.7)	Inactivated	Injection	Turbot	Europe
Icthiovac ^®^ STR	Streptococcosis	Inactivated culture of *Streptococcus parauberis* (strains RA-99.1 and AZ-12.1)	Inactivated	Injection	Turbot	Europe
Icthiovac ^®^ VR	Vibriosis	Inactivated culture of *Listonella (Vibrio) Anguillarum* (serotypes O1, O2a, and O2b)	Inactivated	Injection Immersion	Turbot	Europe
**FATRO (https://www.fatro.it/en/46-fish) (accessed on 20 June 2024)**						
Bi-Fishvax ^®^	Enteric Red Mouth Vibriosis	Inactivated cultures of *Listonella (Vibrio) anguillarum* and *Yersinia ruckeri*	Inactivated	Injection Immersion	Salmonids	Europe
Lacto-Fishvax ^®^	Lactococcosis	Inactivated culture of *Lactococcus garvieae*	Inactivated	Injection	Trout	Europe
Vibri-Fishvax ^®^	Vibriosis	Inactivated cultures of *Listonella (Vibrio) anguillarum* and *Vibrio ordalii*	Inactivated	Injection Immersion	Salmonids Sea Bass Flatfish Ayu	Europe Asia
Yersi-Fishvax ^®^	Enteric Red Mouth	Inactivated culture of *Yersinia ruckeri* (Hagerman type 1 strain)	Inactivated	Injection Immersion	Salmonids	Europe
**Chinese Aquaculture Market** [79]						
(2017) 270446033	Edwardsiellosis Vibriosis	Recombinant antibodies carrying *Edwardsiella tarda* and *Vibrio* sp. idiotypes	Subunit	Injection	Marine Fish	Asia
(2016) 110576037	Edwardsiellosis	Live bacteria culture of *Edwardsiella tarda* (strain EIBAV1)	Live Attenuated	Injection	Turbot	Asia
(2014) 190026031	Grass Carp Hemorrhagic Disease	Live Grass Carp Reovirus (strain GCHV-892)	Live Attenuated	Injection	Carp	Asia
(2011) 190986013	Motile Aeromonas Septicemia	Inactivated culture of *Aeromonas hydrophila*	Inactivated	Injection Immersion	Freshwater Fish	Asia
Cell-cultured inactivated Grass Carp Hemorrhagic Disease Vaccine (1992)	Grass Carp Hemorrhagic Disease	Inactivated culture of Grass Carp Reovirus	Inactivated	Injection	Carp	Asia
Genetically engineered live vaccine against vibriosis (2019)	Vibriosis	Genetically attenuated live culture of *Listonella (Vibrio) anguillarum*	Live Attenuated	Injection	Turbot	Asia
Imported vaccine against fish Infectious Spleen and Kidney Necrosis (2014)	Infectious Spleen and Kidney Necrosis	Inactivated Infectious Spleen and Kidney Necrosis Virus	Inactivated	Injection	Marine Fish	Asia

### 3.2. Contemporary Methods of Aquaculture Vaccine Administration

The efficacy of an aquaculture vaccine relies heavily on the judicious selection of the route and method of administration. A successful vaccine is based not only on its design, but also on its correct and effective administration. Thus, precise application is paramount to ensure optimal immune responses in aquatic organisms. Factors such as the technology of the vaccine, the species and the reproductive stage of the aquatic organism, knowledge on the nature of a pathogen and its routes of infection, as well as the cost require tailored vaccine-delivery strategies to be adopted during the development and implementation of an aquaculture vaccine [74,80].

Even though a vaccine is designed to provide adequate immune responses, the inappropriate or incorrect administration of aquaculture vaccines often fails to yield the intended protective effects against aquatic pathogens [80]. Moreover, careful consideration should be given to practical and logistical aspects, since some methods are more feasible, more cost effective, and less stressful to aquatic organisms. By recognizing the importance of the method of administration, the industry of aquaculture can potentially enhance the overall effectiveness of vaccines and contribute to the overall sustainability of production. Below, the main methods of vaccine administration currently used in aquaculture are presented, and an evaluation based on recent literature information is provided in Table 2.

#### 3.2.1. Injection Vaccination

Most aquaculture vaccines are administered via injection, which can either be intraperitoneal (IP) or intramuscular (IM). This method constitutes an essential strategic approach employed to ensure the precise and controlled delivery of vaccine antigens inside the host organism. Intraperitoneal injections provide the most adequate and long-lasting immunization compared to the rest of the methods [74,81,82]. By bypassing the natural barriers that may potentially impede with the uniform distribution and absorption of the vaccine, injection vaccination ensures the accurate administration of the vaccine antigens in precise doses, while it simultaneously enables the use of adjuvants to boost the process of immunization [73].

Despite its popularity, however, this method of administration does not come without drawbacks. A notable drawback of injection vaccination lies in its potential to induce severe stress to aquatic organisms during vaccine administration. To properly administer injectable vaccines, the aquatic organisms must first be immersed inside an anesthesia solution and then be injected using a vaccine pistol, a tool that enables aquaculture workers to vaccinate up to a thousand individuals per hour [74,82]. It is evident that anesthesia, physical handling, and the injection of the organisms can, in some cases, lead to trauma, potentially compromising the health and safety of the aquatic animals.

Additionally, this process is rather labor intensive. The need for skilled personnel to execute the injections correctly often raises concerns about the cost effectiveness and the practicality of this method, especially in large-scale aquaculture operations. Vaccination through IP injection is also restricted by the organism’s size (usually > 15 g), meaning that smaller organisms like fish fry are excluded, despite their urgent need for immunization. This can prove particularly alarming, especially when taking into consideration that it is during this stage of production that the biggest and most severe losses in finfish aquaculture occur [82].

Following this motif, intramuscular injection is not as popular a vaccine administration method as IP injection, despite being preferred by most aquaculture workers. Consequently, it is mainly recommended for DNA vaccines and is known to induce severe stress and trauma. IM injection is often linked to high mortality rates in aquatic organisms [15,74,78]. All the abovementioned drawbacks hint towards a careful consideration of alternative methods of vaccine administration in order to strike a balance between precision, cost effectiveness, and the welfare of aquatic animals, in large-scale industrial aquaculture operations. For this reason, automated machine vaccination is already being implemented for injection vaccination in fish to minimize some of the disadvantages of manual vaccination [74,81]. As fully automated vaccination machines are commercially available already, this marks another pivotal milestone in aquaculture and adds a compelling alternative for the industry that will only improve as the years go by, thanks to the continuous advancing of technological expertise.

#### 3.2.2. Immersion Vaccination

Immersion vaccination constitutes one of the two alternatives to injection in aquaculture, standing as a methodological cornerstone for improving the immune resilience of aquatic organisms. It is widely used in large-scale aquaculture operations, minimizing the need for animal handling and enabling aquaculture workers to vaccinate large numbers of fish simultaneously, without causing severe stress, as is the case of injection vaccination. Consequently, it is a scalable and rather effective method to implement in industrial settings, an element that is essential for large producing operations [73,74,75,82].

During immersion vaccination, aquatic organisms are submerged inside a formulated vaccine solution, and the antigens are taken up through the mucosal surfaces of the skin, the gills, the intestine, and the nasal cavity [83]. The mucosal surfaces of the skin and the gills constitute the main areas of antigen uptake; however, all mucosal areas are subjected to the vaccine solution during the immersion [84,85]. This stimulates both systemic and localized mucosal immune responses, mimicking the natural routes of infection and promoting a collective defense network against prevalent aquatic pathogens [81,86]. Additionally, immersion enables the vaccination of aquatic organisms regardless of their size, meaning that it can be implemented on smaller organisms as well, such as fish fry [73,74,75,82]. In practice, this is the method of choice for vaccination of fish this size.

**Table 2:** Evaluation of the current vaccine administration methods implemented in aquaculture according to the latest literature information [74,81,82,84,85,86,87].

	Injection Vaccination	Immersion Vaccination	Oral Vaccination
**Immunopotency**	High	Medium	Low
**Practicality**	Low	Medium	High
**Safety**	Low	Medium	High
**Stress Induced**	High	Medium	Low

There are several different ways that immersion vaccination can be implemented in an aquaculture setting. The most common usually differentiate between the time of submersion and the concentration of the vaccine solution used. Bath vaccination involves the controlled immersion of large aquatic organism populations in vaccine solutions for a prolonged period of time, spanning from one to several hours. Alternatively, the organisms can be dipped inside a vaccine solution for a shorter period of time, typically ranging from 20 to 30 s, or be submerged in a more diluted solution for a longer time. The former, often referred to as “Dip Vaccination”, is held in high regard within the industry of aquaculture as a minimally invasive method of vaccine administration that alleviates a considerable amount of stress from the animals. It is overall practical, fast, and rather cost effective at the same time [74,83,87].

Many alternative variations of immersion vaccination have been developed, particularly to maximize antigen uptake and optimize the conferred immunization. It is noteworthy that while immersion vaccination is scalable and efficient, challenges persist due to the relatively weak and brief immunization effects induced [74,87]. This often necessitates several booster vaccinations to be carried out, leading to large amounts of vaccine solutions being used to provide adequate and long-lasting protection [88]. This is precisely why several advancements and experimentations have been conducted to enhance the immunization potency profile of immersion vaccination. These attempts, however, have yielded varying results to date. The most notable examples include the use of spray vaccination [79,81], hyperosmotic infiltration [82,89,90,91], microbubble treatment [92], low-frequency ultrasound sonophoresis [93,94], and stamp vaccination, with the utilization of puncturing instruments [95]. However, all these methods have proven to be rather stress-inducing to animals and require specialized handling by trained personnel, ultimately restricting these advances strictly to experimental settings. In practice, today, the augmentation of immersion vaccination in the aquaculture sector relies heavily on the use of specific adjuvants that further induce the immune response of this method.

#### 3.2.3. Oral Vaccination

The last and most promising alternative to the golden standard of injection vaccination in aquaculture encompasses the oral administration of vaccines to aquatic organisms, collectively known as oral vaccination. This method of vaccine administration boasts quite a number of advantages in comparison to injection and immersion vaccination, often being regarded as the most desirable method of administration amongst workers in the aquaculture sector due to its simplicity and practicality. This method enables the mass vaccination of large animal numbers without the need for any specialized handling. In practice, this minimizes the cost of intensive labor and alleviates animals of stress or trauma, protecting the overall welfare and wellbeing of the organisms reared. In addition, it enables the vaccination of animals of any size, leaving space for a wide range of applications, including the vaccination of fish fry that are often in urgent need of immunization [16,74,79,80,84,96,97].

Conventionally, the oral administration of aquaculture vaccines is carried out either by the direct incorporation of vaccine antigens into the animal feed or simply by mixing [73,74]. It is important to note that most aquaculture feed is produced by means of extrusion, under high levels of temperature and pressure. It is paramount, therefore, that the incorporation of antigens occurs during later stages of feed production, typically after the extrusion and drying of feed, to ensure the integrity of the antigens. The vaccine antigens can be sprayed under vacuum to maximize absorption into the feed, if they are in liquid form, or they can be incorporated with the assistance of adhesive substances, such as edible oils with a high dietary value, if they are in powder form. While straightforward, however, these methods do not guarantee the uniform and homogenous distribution of vaccine antigens into the animal feed [98].

Oral vaccination in aquaculture is typically faced with a wide spectrum of challenges, despite its evident promise. The integrity of vaccine antigens is paramount to this method of administration, as oral vaccines need to be capable of overcoming both the aquatic and the harsh environment of the animal’s digestive tract. The degradation of antigens by low pH values and proteolytic enzymes constitutes an important hurdle to the consistent uptake of oral vaccines, hence adding an additional layer of difficulty to the implementation of oral vaccination in aquaculture settings [97,98,99]. Furthermore, the unpredictable feeding behavior of many species can potentially undermine the consistent and accurate uptake of oral vaccines, eventually restricting this route of administration to mainly booster vaccination applications as of now [73].

All in all, the immunization elicited by oral vaccines is not considered a robust one, especially when compared to injectable alternatives [74,99]. This is further burdened by the phenomenon of oral tolerance, which constitutes a homeostatic mechanism of the immune system that is conserved in teleost fish. This phenomenon confers a tolerogenic effect to ingested elements, essentially safeguarding against any unsolicited immune reactions that might prove harmful during the process of feeding [98,99,100].

To optimize the uptake of oral aquaculture vaccines and induce stronger and longer-lasting immunization, many innovations have emerged, with particular emphasis being placed on encapsulation techniques to address the vulnerable integrity of oral vaccine antigens [97,101]. This has proven quite a verdant field for scientific and technological exploration, as many variables that influence the effectiveness of this approach require elucidation to have a comprehensive grasp on the intricacies that lead to adequate immunization [18]. Current insights reveal that factors like the antigen composition, the vaccine dosage, the water temperature, the species of the aquatic organisms concerned, and the encapsulation technology are all pivotal in attaining adequate immune protection against pathogens via oral vaccination [16,18,79]. Unravelling the interplay between these elements can ultimately advance the understanding of oral vaccination, refining the process of developing efficacious oral vaccines and paving the way for broader-scale implementation in the aquaculture industry.

### 3.3. The Current State of Adjuvants in Aquaculture Vaccinology

Vaccines are preparations containing antigens that are meticulously designed to elicit an immune response and provide protection against diseases caused by pathogens in host organisms. Apart from the specific vaccine technology and the antigen composition, however, many vaccines are accompanied by compounds known as adjuvants that are formulated specifically to enhance immune responses after vaccine administration. These compounds serve as crucial components to many vaccine technologies, boosting the efficacy of the vaccine and providing adequate levels of protection post-vaccination.

Traditionally, adjuvants were defined as auxiliary agents that improved vaccine potency, signifying a boost to adaptive immune responses, or increased its efficacy, thereby preventing infections and diseases thereof. Over time, however, the field of immunology has evolved to acknowledge the role of adjuvants in augmenting immunization through specific and intricate mechanisms. Currently, these substances are given a refined and clearer definition, as a group of structurally heterogenous compounds that regulate the intrinsic immunogenicity of vaccine antigens [82].

In regard to aquaculture vaccinology, the most common and widespread adjuvants utilized are mineral oil-based emulsions, which are known for their ability to create a depot effect by providing a steady release of antigens and prolonging the stimulation of the immune system. Prime examples of mineral oil-based adjuvants in aquaculture include Freund’s Complete Adjuvant, known as FCA, Freund’s Incomplete Adjuvant (FIA), and Montanides™, which constitute a series of commercially available vaccine adjuvants formulated by SEPPIC [82,102,103]. These adjuvants are often referred to as type 1 adjuvants, according to the two-signal model, due to their capacity to modulate and enhance antigen presentation to APCs by influencing the concentration, distribution, and the overall presentation time of the antigens to the APCs [16,82,104].

Another category that has been frequently employed, thanks to its immunostimulating effects, are aluminum salts, like aluminum hydroxide or aluminum phosphate. These are considered type 2 adjuvants, as they provide necessary co-stimulatory signals during antigen presentation that promote the activation of specific B-cell and T-cell populations [105]. Other type 2 adjuvants used in aquaculture vaccinology encompass the use of β-glucans, specifically β1,3-D glucans, and saponins, which have been shown to stimulate the activation of Th1 and Th2 T-cell subsets upon vaccination. In addition, structural elements of microorganisms such as lipopolysaccharides (LPSs), lipopeptides (LPs), and proteins have also been utilized as adjuvants in aquaculture vaccinology. Prime examples include polar glycopeptidolipids of *Mycobacterium chelonae* (pGPL-Mc) and flagellins of Gram-negative bacteria [82].

For viral vaccines, the implementation of Poly I:C has been reported to stimulate the production of interferons, namely IFN-1. Synthetic adjuvants have also been tested in aquaculture vaccinology, with synthetic CpG oligonucleotides (CpG ODNs) being prime examples, as they are capable of triggering immunostimulating cascades that orchestrate the maturation, differentiation, and proliferation of a wide array of immune cells, like B cells, T cells, monocytes, macrophages, dendritic cells, and NK cells. These motifs are up to 20 times more abundant in microbial DNA than mammalian DNA, making the ODNs carrying them an ideal adjuvant candidate for effective vaccine development. Several studies have also demonstrated their effectiveness in fish vaccination, hinting at their potential as adjuvants to increase the immunogenicity of DNA vaccines used in aquaculture settings [82].

It is important to note that different adjuvants can be combined with different antigens and provide different types and scales of immunostimulation, ultimately leading to totally different immunization outcomes conferred as well [106,107,108]. Thus, the meticulous selection of antigen–adjuvant combinations is pivotal in novel vaccine development, even in the context of aquaculture. This synergy is a cornerstone to vaccine formulation, warranting extensive research and evaluation to maximize efficacy and minimize detrimental side effects. Despite being popular and effective in aquaculture vaccinology, commonly utilized vaccine adjuvants like oil-based emulsions are known to induce unwanted side effects upon fish vaccination, including granulomas, inflammatory reactions, tissue adhesions, and discolorations, leading to lower-quality fish products and compromised animal welfare. As such, ongoing advancements in adjuvant technology hold promise for enhancing the immunogenicity and the protective capacity of vaccines in aquaculture, ensuring that the most suitable combinations of antigens and adjuvants can be validated through research, and new alternatives can arise.

## 4. A Review on Recent Developments and Milestones

In this section, recent developments and milestones for diffuse and different aspects of aquaculture vaccinology are synthetically analyzed to provide a complete and up-to-date understanding of current affairs in global aquaculture vaccine research. Specific focus is given to alternative and upcoming vaccine technologies, new insights and applications on vaccine adjuvants, progress in oral vaccination strategies, and milestones reached in the complex process of developing vaccines for aquatic multicellular pathogens. At the end of this section, a summary of the analysis that includes advantages and disadvantages for every milestone and topic discussed is presented in Table 3.

### 4.1. Alternative and Upcoming Vaccine Technologies for Aquaculture

#### 4.1.1. mRNA Vaccines

mRNA vaccines constitute a relatively novel vaccine technology, having only been developed recently, during the past three decades, as an alternative to DNA vaccines. Despite being challenged with issues regarding their stability and correct application, mRNA vaccines have come under substantial progress in recent years, making their large-scale production economically viable thanks to the scientific and technological advances of biotechnology, immunology, and molecular biology [16,109]. Currently, this technology holds high value as a potential platform for new vaccine development, since mRNA vaccines are distinguished by their increased immunogenicity, safety, and their low production cost [110]. The implementation of this vaccine technology in aquaculture settings holds promise, signifying a revolutionary stride in the realm of aquatic disease prevention in the modern era.

A typical mRNA vaccine consists of all the necessary molecular elements that comprise an mRNA molecule, allowing it to be expressed through the process of translation in the cell’s ribosomes. These include an open reading frame (ORF) for the targeted antigen, situated along 5′ and 3′ untranslated regions (UTRs), a 5′ cap, and a terminal poly(A) tail [109,111]. After administration, the mRNA vaccine goes through the process of translation in the cell’s ribosomes and is expressed into the antigen of interest, following the fundamental principles of molecular biology. It is worth noting that a single mRNA molecule only encodes a specific antigen, though the very same molecule can be expressed to produce a significant number of antigens. This potential, however, is limited, as mRNA molecules are prone to enzymatic degradation inside cells [111].

Conventional mRNA vaccines do not self-amplify inside the host’s cytoplasm. There do exist, however, self-amplifying mRNA vaccines, based on the bioengineered genomes of viruses, called replicons. These constructs utilize the self-amplifying machinery of recombinant viruses, while simultaneously replacing the functional and structural viral proteins with antigenic gene sequences. Consequently, this means that replicon-based mRNA vaccines can mimic viral infections and provide robust humoral and cellular responses without being infectious or posing any safety risks, since viral particles cannot be formed in the absence of the abovementioned functional and structural viral proteins [15,109,111].

The most common viruses utilized for this technology belong to the sole genus of the Togaviridae family, called *Alphavirus*. Alphaviruses are positive-sense, single-stranded RNA viruses known to cause a wide range of infectious diseases, both in vertebrates and invertebrates. They are particularly known for diseases transmitted via arthropods, hence being categorically encompassed under the informal collective umbrella term of arthropod-borne viruses, known as arboviruses [112]. Alphaviruses hold immense potential as platforms for novel vaccine development, especially in aquaculture, since many members of the genus cause diseases in economically important fish species.

Salmonid alphaviruses (SAVs) are significant viral pathogens of the aquaculture industry, sharing a considerable percentage of their genome with mammalian alphaviruses. This constitutes a sound base for scientific exploration and novel vaccine development, as there already exist commercial mRNA vaccines for mammalian production animals in the U.S.A. today [15]. One of the most notable applications of SAV-based replicon vaccines for aquaculture can be found through Wolf et al.’s work, where an effective vaccine against the infectious salmon anemia virus (ISAV) was developed [113]. This vaccine, based on an SAV-3-based replicon encoding the hemagglutinin-esterase (HE) protein of ISAV, was proven to be capable of providing adequate protection against infectious salmon anemia (ISA) when administered through IM injection. This study also cemented the immunogenic properties of HE, as neither the matrix (M) nor the fusion glycoprotein (F) proteins were noted to be essential for immunization against ISA.

These findings suggest that the mRNA vaccine technology holds promising value as a potential candidate for novel aquaculture vaccine development, though attempts in this realm still appear to be in their infancy. Consequently, this highlights the need for further optimization through extensive trials and dedicated research efforts to firmly establish mRNA vaccines on the landscape of future aquaculture vaccine technologies. As researchers continue to explore the efficacy of mRNA vaccines in aquatic organisms, collaborations between academia, industry, and regulatory bodies will be essential to expedite their integration into mainstream aquaculture practices in the foreseeable future.

#### 4.1.2. Vector Vaccines

Vector vaccines utilize living, non-pathogenic microorganisms in general, as carriers for the effective transportation of vaccine antigens inside the host [72,114]. Known for its ability to combine the immunogenicity of live-attenuated vaccines with the high precision of subunit vaccines, this vaccine technology constitutes a noteworthy alternative for vaccine development, exhibiting many advantages in its ability to provide high levels of protection through the elicitation of specialized immune responses [76,115]. During the last couple of decades, numerous attempts have been made to advance the development of vector vaccines in the aquaculture industry. By utilizing vectors that are no longer attenuated through traditional chemical or physical means, but rather through contemporary genetic engineering, virulent genes can now be deleted or be replaced with potential genes of interest, opening new gateways for efficient and safe vaccine production [76].

As far as bacterial vectors are concerned, many bacteria like *Listeria monocytogenes* and *Escherichia coli* have been efficiently employed for the development of aquaculture vector vaccines against economically important diseases such as vibriosis [116,117]. Spore-forming bacteria like *Bacillus subtilis* have also been utilized for the development of vector vaccines against a wide array of aquatic diseases, namely streptococcosis [118], reoviral infections [119], and even cercarial parasitic infections caused by the trematode platyhelminth *Clonorchis sinensis* [120]. Within the same realm, *Lactobacillus casei* and *Lactococcus lactis* bacteria have been successfully utilized for the development of vaccines against *Aeromonas veronii* [121,122] and the viral hemorrhagic septicemia virus (VHSV) [123], respectively.

In the domain of viral vectors, recombinant baculoviruses have emerged as effective tools for developing viral vector aquaculture vaccines, particularly against emerging viruses like the Cyprinid herpesvirus 2 (CyHV-2) [124], VHSV [125], and the infectious spleen and kidney necrosis virus (ISKNV) [126]. As baculoviruses are known to infect invertebrates, this platform can also be utilized to vaccinate economically important species of aquatic arthropods against severe and detrimental diseases, such as the white spot syndrome (WSS) and nodaviral infections [127,128,129,130]. Nevertheless, baculoviruses can integrate their genomes in the host’s chromosomes, thus making their commercial application for vaccine development a near impossible task for aquaculture. This genome insertion can potentially cause cancer onset, in the same scope of retroviral or lentiviral infections, through a phenomenon called oncogenic insertion. Additionally, a random genome insertion could make the vaccinated animals be legally regarded as genetically modified organisms (GMOs), meaning there are severe repercussions in terms of safety, consumer acceptance, and legality, especially in areas with strict GMO legislations [99]. Hence, the quest for effective viral vector vaccines has extended to alternative vector paths, most notably recombinant adenoviruses. These viruses exhibit a strong safety and versatility profile as viral vectors, having emerged as an appealing platform for the advancement of viral vector vaccines in veterinary science for a wide range of species and diseases [131,132].

Recently, Ling et al. successfully implemented the recombinant adenovirus platform to develop a viral vector vaccine against the bacterial pathogen *Aeromonas salmonicida* in rainbow trout (*Onchorrynchus mykiss*) [133]. In the same year, Li et al. developed a recombinant adenoviral vaccine against infectious hematopoietic necrosis (IHN) caused by the IHNV virus [134]. The latter also developed a similar vaccine against the infectious pancreatic necrosis virus (IPNV), ultimately advancing their research to make a bivalent recombinant adenovirus vaccine that provided adequate protection against both viruses [135]. These recent breakthroughs indicate that despite being in its nascent stages as far as the realm of aquaculture is concerned, the recombinant adenovirus platform can indeed be used to efficiently develop viral vector vaccines. This platform has even demonstrated the capability of conferring protection against co-infections in economically significant species, marking significant advancements in vaccine development. This highly promising outcome is poised to usher in new paths to the implementation of alternative vaccine technologies in aquaculture, contributing significantly to the quest for aquatic disease prevention and to modern sustainability efforts.

Apart from live vectors, however, synthetic vectors have also been harnessed for the development of vector vaccines in the aquaculture industry. This dynamic shift has prompted the global scientific community to differentiate between living and non-living vaccine vectors, a differentiation that has become even more pronounced in recent years, thanks to the rapid evolution of bioengineering and nanotechnology. Within the realm of aquaculture, two candidates hold promise as synthetic vectors for vaccine development, those being virus-like particles (VLPs) and bacterial ghosts (BGs).

Virus-like particles are self-assembling molecular structures that essentially emulate viral particles. Made out of viral capsid proteins devoid of any original genetic material, these particles are rendered non-infectious but are still able to elicit immune responses by mimicking virus assembly at a tertiary level of structure [136]. Virus-like particles can be produced through heterologous expression systems, such as bacteria, yeasts, mammalian or insect cell lines, and even transgenic plants [15,136,137]. Thanks to their properties, they have been explored for their potential as vectors in vaccine development, since they are capable of eliciting both humoral and cellular immune responses upon administration [15]. According to recent reviews carried out by Jeong et al. and Angulo et al., the main body of VLP vector vaccines in aquaculture stems from the utilization of nodaviral VLPs, specifically those of the *Betanodavirus* genus [136,138]. This platform has been employed to develop VLP vaccines against viral nervous necrosis, a fish disease also known as viral encephalopathy and retinopathy (VER), caused by the betanodavirus NNV. NNV–VLPs appear to be an ideal platform for aquaculture vaccine development, and their development can be facilitated through a multitude of expression systems [139]. As a result, NNV–VLP vaccines have been developed not only to confer protection against nodaviruses but also against other pathogens of viral origin such as IPNV, VHSV, SAVs, and iridoviruses.

The NNV–VLP platform was recently utilized for a potential vaccine against the bacterial pathogen *Streptococcus iniae*. The VLPs displayed the bacterium’s α-enolase on their surface and were able to reduce the mortality in olive flounder (*Paralichtys olivaceus*) and in zebrafish (*Danio rerio*) upon preliminary immunization [140]. It is important to note that VLP vaccines are very safe; recent studies have shown that they do not evoke any clinical side effects but elicit extensive immune responses. This includes the upregulation of both innate and adaptive humoral and cellular components, as observed recently in a gene expression analysis conducted in European sea bass (*Dicentrarchus labrax*) [141].

On the other hand, BGs are products of chemical or biological processing of partially lysed bacterial cells, resulting in husks that retain their morphological integrity and structural immunogenic components, though they are devoid of any intracellular contents. By preserving the bacteria’s inherently immunogenic structural components, like LPS, lipoproteins (LPNs), and peptidoglycans (PGNs), BGs can act as immunostimulatory vaccine carriers and be detected by receptors that recognize PAMP motifs [142,143]. Gram-negative bacteria are often utilized for the development of BGs, forming an interesting platform for synthetic vaccine carriers by also providing built-in adjuvant effects. According to Zhu et al.’s latest review on BG vaccines, it appears that this platform has gained a newfound interest recently for the development of aquaculture vaccines, as BG vaccines have been shown to attract momentum in the realm of aquaculture vaccinology. Bacterial ghost vaccines have been reported and developed against a multitude of aquatic pathogens, including bacterial pathogens belonging to the genera *Edwardsiella*, *Aeromonas*, *Flavobacterium*, and *Vibrio*, as well as viral pathogens like the grass carp reovirus (GCRV) and the spring viraemia carp virus (SVCV) [142].

#### 4.1.3. Synthetic Peptides—Epitope Vaccines

Synthetic peptides or epitope vaccines can be considered an improvement of conventional subunit vaccines, as they contain short, synthetically manufactured amino acid sequences that are designed to be maximally immunogenic and induce strong immune responses upon administration as vaccine antigens. Until recently, this technology was not considered to be practical for vaccine development purposes, since there was limited comprehensive insight into the immunogenicity of antigens and their interplay with the host’s immune system [73]. However, thanks to the recent advances of computational biology, synthetic peptide vaccines can now be successfully developed, because the exact immunogenic determinants called epitopes can be mapped extensively using in silico bioinformatic approaches. Therefore, vaccines carrying synthetic peptides that are structurally identical to pathogen epitopes can be created, forming a promising novel platform for vaccine development, even in aquaculture.

Despite this technology not being extensively implemented in aquaculture vaccinology yet [72,73], several attempts have been recently made to explore the epitopes of many aquatic pathogens. In 2016, Mahendran et al. mapped potential epitope candidates for the development of synthetic peptide–epitope vaccines against the bacteria *Edwardsiella tarda* and *Flavobacterium columnare* [144]. In the same year, Sharma and Dixit explored a recombinant chimeric epitope for the bacterial pathogen *Aeromonas hydrophila* [145]. This sparked a cascade of publications, as Baliga et al. identified potential epitopes for *Vibrio anguillarum* [146], and Pumhcan et al. developed a chimeric multiepitope vaccine against streptococcosis in Nile tilapia (*Oreochromis niloticus*) [147].

It is rather encouraging to witness the implementation of increasingly sophisticated methodologies being used in the pursuit of epitope identification and the development of aquaculture vaccines, as recently, in 2022, Islam et al. explored an immunoinformatic and integrated core proteomic approach to identify and develop epitope vaccines against *Edwardsiella tarda* and *Aeromonas veronii* [148,149]. In the realm of viral pathogens, attempts have been primarily focused on betanodaviruses [150,151,152,153], iridoviruses [154], and the tilapia lake virus (TiLV) [155,156]. The exploration efforts have also similarly been extended towards the direction of aquatic invertebrate vaccine development, as demonstrated by the endeavors of Momtaz et al. [157], Shine et al. [158], and Islam et al. [159].

The pursuit of epitope identification and the optimization of vaccine development for important aquatic pathogens like *Edwardsiella sp.* and *Flavobacterium columnare* are still ongoing, as one can see by the more recent publications on the topic [148,160,161]. This serves as promising proof that the technology of synthetic peptide–epitope vaccines is a valuable tool for aquaculture vaccine development and is expected to further improve in the coming years with the current onset of -omics approaches. These approaches are bound to facilitate the establishment of an integrated framework, where the study of aquatic organism immunology and host–pathogen interactions will enable the development of next-generation vaccines, in collaboration with computational and structural biology. The latest advent of artificial intelligence (AI) technologies holds potential to contribute equally to this pursuit, by further enhancing the relatively nascent field known as reverse vaccinology within the context of aquaculture vaccinology as well.

### 4.2. Reverse Vaccinology

Reverse vaccinology is not only a relatively new-coined term but also a burgeoning new field in vaccinology, referring to the collective process of identifying suitable antigen candidates through the comprehensive study of pathogen genomes [16]. Essentially, it encompasses a predictive bioinformatic genomic analysis that pioneered its implementation in the early 21st century, a trajectory that was set in motion by the worldwide trend following the first successful genome sequencing of a microorganism in 1995 [162,163,164,165]. By studying the entire genome of a pathogen, and thus its proteome, algorithms can now be employed to identify the most promising and suitable antigen candidates, which can then be produced using the means of biotechnology. Effectively, this reduces the total production time of vaccine development from 5–10 years to only 1–2 years, according to recent estimates [74,166].

In the realm of aquaculture vaccinology, this technology has been increasingly implemented in the development of novel vaccines, as it can be observed through the previously mentioned studies on epitope identification and synthetic peptide–epitope vaccines. Today’s level of technological competence enables the implementation of high-throughput sequencing (HTS) techniques, meaning that the most potent and suitable antigens for vaccine development can now be identified with increased precision and specificity. Recently, Chukwu-Osazuwa et al. presented an extensive list of potential antigen candidates for many prevalent bacterial pathogens in Atlantic salmon and lumpfish aquaculture, using a comparative reverse vaccinology approach [164]. As such, a foundation for the effective and fast-track development of polyvalent aquaculture vaccines is already being set in motion. Common antigens between pathogens can now be identified and be incorporated in vaccines that will confer cross-protection against multiple pathogens simultaneously. This is a pivotal step for the aquaculture industry, as high-density and intensive rearing is a reality, increasing the danger of co-infections exponentially.

Contemporary bioinformatic tools, coupled with the utilization of predictive mathematical models, can currently enable the simulation of immune system dynamics and its responses during infection across a diverse array of species. In 2017, Madonia et al. published a study in which they reported the simulation of the immune response of sea bass against the bacterial pathogens *L. anguillarum* and *P. damselae* [167]. This was achieved through the implementation of a computational model equipped with the species’ main immunological attributes. The predictive power of the model was later assessed using the results from two in vivo vaccination trials. Ultimately, this led to the conclusion that the model can be efficiently utilized for the optimization of fish vaccination, going as far as predicting the level of immunization by factoring in several parameters, such as the vaccine dosage and route of administration, for multiple pathogens simultaneously. This can only be seen as an invitation to utilize computational methods for a wider range of aquatic species and pathogens, thus paving the way for the adoption of cutting-edge automated technologies in aquaculture vaccination.

The suitability of in silico methods for aquaculture vaccine optimization is bound to be boosted by the recent advent of AI and the field of systems biology, especially after the most recent COVID-19 pandemic [168,169]. Machine learning (ML) can prove to be a valuable tool for the automatization and the optimization of computational models and their predictive capabilities. Ultimately, this can facilitate the rapid development of highly specialized vaccines, with increased efficacy in the future of aquaculture [170]. These developments are predicted to significantly influence typical vaccine manufacturing processes, heralding a new era for aquatic disease management, among others.

### 4.3. Recent Adjuvant Breakthroughs in Aquaculture Vaccinology

Nowadays, the aquaculture industry is witnessing two cutting-edge trends dominating the realm of adjuvant technology worldwide. These innovations pertain the utilization of nanoparticles and the exploration of cytokines, both aimed to enhance the efficacy of immunization upon vaccination. In the coming segments, both will be analyzed in respect to the recent attention they have garnered as aquaculture vaccine adjuvants. Additionally, specific studies showcasing innovations will be highlighted, always in the context of fish immunology and vaccination.

#### 4.3.1. Nanoparticles as Aquaculture Vaccine Adjuvants

Nanoparticles (NPs) are small particles ranging from 1–100 nm in size, offering a versatile vaccine delivery and adjuvant platform thanks to their diminutive size and unique physicochemical properties. The most common category of NPs employed in aquaculture vaccines currently include nanopolymers, particularly because of their biocompatibility and biodegradability inside the hosts [171]. They are usually employed as effective vaccine carriers in oral vaccination, offering extra immunogenic properties as adjuvants, as is the case with chitosan nanopolymers and synthetically derived Poly (lactic-co-glycolic acid) or PLGA nanopolymers [172]. Lipid-based nanoparticles are also used as synthetic vaccine carriers, offering the ability to augment immune responses when effectively combined with an adjuvant to form a polymer “vehicle” for targeted antigen transportation. This technology is often referred to as immune-stimulating complexes (ISCOMs), which encompass the implementation of self-assembling structures of around 40 nm that are made of saponins and lipids, such as cholesterol or phospholipids [171].

Inorganic nanoparticles have also emerged as promising adjuvant candidates in recent developments, thanks to their physical and chemical properties. Their compact structure, as well as their ability to protect and provide targeted antigen transportation to the host, make them well-suited carriers with strong adjuvant properties for vaccine development [171]. A most recent example, as far as aquaculture vaccinology is concerned, is the implementation of carbon nanotubes, known as CNTs. These structures provide an interesting adjuvant platform, since they are non-toxic, and enable the delivery of antigens to APCs with increased efficiency, thanks to their large surface area and their mechanism of entry inside the host’s cells [173,174]. The use of CNTs as adjuvants in aquaculture vaccinology has been extensively documented, providing not only proof of their value as future vaccine adjuvants but also insights into their immunostimulating effects in both injection and immersion fish vaccination [175,176,177,178,179,180,181].

#### 4.3.2. The Adjuvant Activity of Fish Cytokines

On the other hand, the adjuvant and immune-enhancing capacity of cytokines in mammals is highlighted in an abundance of existing literature, though exploration in the context of fish immunology remains sparse. As such, recent publications have embarked on a journey to elucidate the exact adjuvant effects of cytokines in fish, as pointed out by Guo and Li in their recent review on fish cytokines [182]. Cytokines are proteins secreted by a plethora of immune cells that help modulate immune responses through the regulation of intricate signaling pathways. One of the main cell types responsible for cytokine production are Th cells, where each subset is known to secrete different types of cytokines. In terms of fish immunology, the study of three types of cytokines has recently gained interest in terms of scientific exploration. Namely, these three types of cytokines consist of Th0, Th1, and Th2 cytokines.

As far as the first category is concerned, IL-1β appears to have adjuvant properties due to signaling effects on inflammation and antibody production, whereas IL-8 is hinted to regulate a wide array of immune responses upon vaccination, depending, however, on the type of vaccine technology used [183,184]. The adjuvant properties of IL-12 in fish immunology have also been highlighted in recent literature by Matsumoto et al. [185], alongside IL-15 and IL-17, which have been shown to enhance antiviral and cellular immune responses [181]. The granulocyte colony-stimulating factor (G-CSF), although not extensively explored in fish, also appears to exhibit adjuvant properties to some extent. Lastly, studies focusing on the role of TNF-α in the context of fish immunology have also been explored, with results on its adjuvant properties remaining controversial so far [182].

In terms of Th1-secreted fish cytokines, interferons seem to monopolize the interest of scientific endeavors, with results clearly highlighting the adjuvant properties of IFN-α, IFN-c, and IFN-γ [186,187]. Nevertheless, IL-2 is also a Th1 cytokine that has been shown to have adjuvant effects, boosting humoral, cellular, and inflammatory immune responses when administered with DNA vaccines [182,187]. The same result has been observed with the administration of vaccines containing antigens of a protein nature [188]. In the context of Th2 cytokines, IL-6 was recently shown to have adjuvant properties upon vaccination against bacterial pathogens when administered with both subunit and DNA vaccines [189,190]. Similarly, the immune-enhancing properties of CC or β-chemokines containing an N-terminal CC domain was recently highlighted, as it was shown that they can enhance the specificity of an immune response through the attraction of different kinds of immune cells, such as B cells, T cells, and DCs [182].

### 4.4. Progress in Oral Vaccination

Oral vaccination still holds the reins as the most attractive route of vaccine administration in the aquaculture sector. To address the challenges that are typically associated with the application of this method, concerning the integrity and availability of vaccine antigens inside the harsh conditions of the gastrointestinal tract and the aquatic environment of fish, encapsulation has emerged as a predominant technology in the production of effective oral vaccines in the industry of aquaculture [97]. Typically, this approach encompasses the utilization of polymers, which are either natural or synthetic in nature. Traditionally, these strategies have mainly been implemented using chitosan, alginate, and PLGA polymers. However, recent advances are witnessing the utilization of increasingly more cutting-edge technologies for this purpose.

#### 4.4.1. Polymer Encapsulation

Chitosan is a naturally derived polymer, stemming from a polysaccharide that is extracted from the processing of arthropod chitin exoskeletons, usually by deacetylation under alkaline conditions [191]. The main advantages of chitosan are predominantly focused on its biocompatibility, its low toxicity, and its biodegradability. Additionally, chitosan exhibits potent mucoadhesive properties which are pivotal for its adjuvant effects. The production of this polymer is neither financially, nor technically demanding, thus making it an ideal carrier for the transportation of vaccines inside organisms. Its efficacy is highlighted by its extensive use in oral vaccination, having been combined with a plethora of different vaccine technologies successfully, as stated in both Dalmo et al.’s and Wu et al.’s recent reviews [82,191].

Alginate polymers on the other hand are derived from the processing of brown algae and are extensively used for the encapsulation of oral vaccines in aquaculture. They provide a low-budget, non-toxic alternative, with strong mucoadhesive and adjuvant properties that have been highlighted in many recent publications [192,193,194]. Primarily, these polymers are used in the form of polymeric globules, often collectively referred to as “alginate beads”.

Lastly, PLGA polymers have also been utilized for the encapsulation of aquaculture vaccines, as stated by Dalmo et al. [82]. These polymers encase vaccine antigens in biodegradable particles, whose biodegradation rate can be determined and modified in advance. Essentially, this offers the advantage of providing a predetermined constant and steady-paced release of antigens that aids antigen presentation to APCs [82,195,196]. It is worth stating that in a recently published study, chitosan polymers were combined with PLGA polymers for the effective transport of oral vaccines in fish [193,194]. The combined use of these two polymers was shown to elicit increased levels of immunization against bacterial pathogens in fish, ultimately providing sound evidence for a future implementation of combined-polymer approaches in oral aquaculture vaccination.

The rapid integration of nanoscale carriers for oral vaccine delivery is gradually making its presence in aquaculture [97,138]. Nanoparticles allow for the highly specific and targeted delivery of antigens inside hosts, while providing immune-enhancing effects due to their adjuvant properties [197]. This combination encompasses a rather promising platform to boost the efficacy of oral vaccines in the aquaculture industry, with the potential of negating any detrimental side effects of traditional adjuvants such as oil-based emulsions [82,198,199,200,201,202,203,204]. The nanoscale utilization of vaccine carriers can not only involve polymers like chitosan, alginate, and PLGA particles but also inorganic nanoparticles, nanoliposomes, nanogels, nanoemulsions, and VLPs.

#### 4.4.2. Bioencapsulation

Bioencapsulation is another interesting and rather promising encapsulation strategy currently explored in the field of aquaculture oral vaccination. This novel approach involves the utilization of living organisms as biological carriers for the delivery of vaccine antigens, usually resorting to organisms that are typically used as feed in aquatic organism nutrition [205]. By ingesting these organisms, the bioencapsulated antigens are absorbed intact, facilitating the stimulation of both systemic and mucosal immune responses [97]. This platform enables the oral vaccination of smaller-sized aquatic organisms, with fish fry being placed in the center of this approach, as it allows vaccine antigens to be incorporated in planktonic organisms that serve as vital food sources for the fry and are rich in polyunsaturated fatty acids (PUFAs), pigments, and antioxidants [84,206,207,208]. The main organisms utilized in the bioencapsulation of vaccine antigens are mainly crustaceans of planktonic nature, such as brine shrimp nauplii of the genus *Artemia*, water fleas of the genus *Daphnia* for freshwater applications, and various small rotifers [74,97]. Overall, bioencapsulation has been utilized in oral vaccination strategies against both viral and bacterial aquatic pathogens, with results proving quite encouraging so far [74]; however, there is still discussion revolving around their ability to reach deep into the lymphoid tissues and elicit long-term protection [209].

Another group of microorganisms that can be utilized in various bioencapsulation approaches for oral vaccination applications in aquaculture are yeasts, since they provide both rich nutrients and immunostimulating effects upon ingestion by aquatic organisms [207]. Recombinant yeasts can also be incorporated inside other organisms, like plankton, to express and deliver antigens inside fish and fish fry. This “Trojan horse” approach offers an interesting platform for protected antigen delivery upon oral vaccination, since it secures the protection of the antigen’s integrity, as long as they are expressed correctly by the recombinant yeast cells inside the planktonic organism.

Recent insights into this strategy are given through Embregts et al.’s published study, where recombinant *Pichia pastoris* yeast populations expressing green fluorescent proteins (GFPs) were successfully bioencapsulated inside *Daphnia* planktonic crustaceans and rotifers that were later fed to fish larvae [207]. Simultaneously, the non-encapsulated form of the same yeast was orally administered to adult fish. The results show the efficient delivery of intact GFPs to the fish larvae’s intestines, proving that the bioencapsulated form of *Pichia pastoris* can be a suitable vehicle for the delivery of oral antigens. Additionally, the adult fish that were fed the non-encapsulated form showed systemic immune responses in their spleens, following the elicitation of swift, localized responses in their intestines. Similarly, Ma et al. explored the utilization of another species of yeast, *Saccharomyces cerevisiae*, as a tool for oral vaccine delivery [205]. In their study, an oral subunit vaccine was developed through the yeast’s surface display of an envelope protein of Cyprinid herpesvirus 3 (CyHV-3). The recombinant antigen-expressing yeast cells were bioencapsulated in *Artemia* nauplii and were subsequently fed to carp larvae. The delivery of the vaccine to the larvae’s hindgut was highlighted, resulting in increased levels of immunization, evidenced by the production of anti-CyHV3 antibodies. These results showcase that bioencapsulating oral vaccines can in fact be exploited in aquaculture vaccinology, offering an important platform and solution to the immunization of fish larvae in the future.

#### 4.4.3. Plant-Based Vaccines

In the dynamically progressing field of oral vaccine development, the utilization of plants has similarly gained traction as a potential avenue of exploration for vaccine development, including in the aquaculture sector. This mirrors global trends that witnessed the development of veterinary plant-based vaccines for a plethora of diseases, such as Newcastle disease in poultry, rabies, and the Porcine Respiratory and Reproductive Syndrome (PRRS). In 2016, the first plant-based vaccine was licensed in the U.S.A., signifying a new milestone for the world of veterinary vaccine development [18,210,211].

Under the doctrine of molecular farming, transgenic plants can serve as an effective platform for vaccine development, specifically bioengineered to produce vaccine antigens in bulk [18]. The utilization of plants constitutes an inexpensive and economically sustainable strategy, since both plant cells and whole plants can be used to produce antigens. Additionally, plants possess the ability of eukaryotic post-translation modifications, boasting a significant advantage over conventional microorganism-based expression systems currently in use [18,74,210,212].

In 2019, Marsian et al. successfully developed a plant-based VLP aquaculture vaccine against VER that provided adequate protection in sea bass against betanodaviral infections [213]. Similarly, Su et al. published their work on the development of a plant-based subunit vaccine against the piscine myocarditis virus (PMCV) [214]. The results show that the developed vaccine induced innate immune responses, reduced the severity of histological findings, and slightly lowered the viral RNA load in vaccinated fish, although the levels of the induced protection were not deemed significant. Collectively, these findings indicate that plant-based vaccines represent a promising avenue for advancing aquaculture vaccinology. Nevertheless, there is a pressing demand to advance the exploration of plant antigen expression systems to ascertain their effectiveness in the future landscape of vaccine development.

The majority of veterinary plant-based vaccines utilize either plant cell lines or edible, whole transgenic plants designed for direct consumption. Common choices include plants that can serve as animal feed, like carrots, potatoes, lettuce, and tomatoes [210]. The latter approach encompassing whole plants has not been utilized in aquaculture yet, though an exciting alternative has recently begun to attract leverage, particularly through the utilization of microalgae. Microalgae are aquatic plants that have gained tremendous interest in scientific and technological exploration during the last decade, serving as a sustainable platform for the production of food, supplements, biofuel, and pharmaceuticals [215,216]. A notable advantage of microalgae lies in their utilization as expression systems to produce vaccine antigens, since microalgae-based systems often exhibit increased efficiency in production compared to the traditional plant-based ones [217].

Recently, Kwon et al. demonstrated that recombinant microalgae of the *Chlamydomonas reinhardtii* species can indeed be utilized for the oral delivery of vaccines, by combining microalgae’s expression capabilities with a bioencapsulation approach in zebrafish [218]. Similarly, Abidin et al. reported the utilization of the microalgal species *Nanochloropsis* to produce vibrio antigens, after optimizing its transformation process [219] and assessing the stability of the expression of the antigen genes in the subsequent transgenic Nanochloropsis generations [220]. Taken together, these studies essentially open the gateway for the further development of transgenic microalgal oral vaccine-delivery systems to be implemented in the aquaculture industry, indicating that plant-based vaccines are potentially attainable in the future of aquatic health management.

It is worth mentioning that the sturdy cell walls protecting the vaccine antigens from the harsh conditions of the gastrointestinal tract, in collaboration with their miniscule size, have propelled the use of microalgal vaccines to applications concerning aquatic invertebrates as well [215,217]. It has already been shown in a past study dealing with crayfish vaccination that transgenic *Dunaliella salina* microalgae can express recombinant WSSV proteins [221]. Since then, studies have managed to elaborate further on the matter, eventually leading to the development of microalgal vaccines based on *Chlamydomonas reinhardtii* that were able to reduce the mortality rate of shrimp against WSS from 100% to just 13–30% [222,223]. Similar developments have also transpired with different species of microalgae, such as *Anabena* and *Synechocystis* [224,225]. Results showcase that the mortality rate of shrimp against WSS can indeed be dropped from 100% to 30–35% by implementing such approaches.

The promising results from all previously mentioned studies signal a positive trajectory for the utilization of plants and microalgae in the development of orally administered aquaculture vaccines overall, affirming that there is substantial room for advancements in the near future. As efforts continue to optimize and explore new expression systems, the production capabilities of such platforms are expected to advance towards more efficient, cost-effective, and sustainable ways. At the same time, the elucidation of the precise and intricate mechanisms behind efficient antigen uptake during oral administration is bound to usher in a notable bloom in the domain of edible oral vaccines, one that will ultimately align with the imperative to develop vaccines that are not only easy to administer but also capable of inducing mucosal immunity in fish and other species of interest.

### 4.5. Recent Progress in the Development of Vaccines against Aquatic Multicellular Pathogens

In the dynamic realm of aquaculture, the unrelentless threat posed by parasitic infections remains a formidable challenge, as it impedes sustainable growth and productivity. Although there is a substantial number of commercially available vaccines against bacterial and viral diseases today, parasite vaccines appear to lag behind in their development and availability, despite parasitic diseases being an important generator of economic losses in the aquaculture industry [226]. The production of parasite vaccines constitutes a challenging endeavor, as parasites are complex multicellular organisms with intricate life cycles and host–pathogen interactions at each developing stage. The dynamic and opportunistic nature of many parasitic infections stands as an additional impediment to potential vaccine development, as it is governed by a multitude of factors, such as the species and the age of the host, the species of the parasite, the infection site, the rearing conditions, and the ambient temperature [227]. Culturing the parasites and establishing a challenge model for screening purposes constitutes an additional challenge, though advances in parasite culture are being made for an increasing number of aquatic parasites, both in vitro and in vivo [228].

Despite the challenges, however, it is worth mentioning that the journey towards developing efficient vaccines against parasites in aquaculture is marked by significant and steadfast progress. The immune responses of aquatic organisms infected with parasites have been well documented for over a century, proving that some levels of protective immunity are indeed present in surviving hosts [229]. This serves as a reminder that the potential of vaccine development against parasites is possible and that the progress of technology is poised to aid this endeavor. Modern tools can contribute to both the elucidation of intricate host–parasite interactions and the production of stable, highly immunogenic parasite antigens in high-producing expression systems [227].

#### 4.5.1. Milestones in Sea Lice Vaccine Development

A frontrunner of parasitic infections in the aquaculture industry is sea lice, especially when taking into consideration the detrimental effects they can have on economically significant fish species like the Atlantic salmon [230,231,232]. Sea lice are naturally occurring ectoparasites of fish, though increasing rearing densities have sparked rather high infestation rates in both farmed and wild fish populations during the last decades. This parasite tends to feed on the skin, the blood, and the mucus of fish, leading to severe stress, anemia, and ulcerations that can cause further infections if left untreated [233]. Additionally, it has been recently highlighted that sea lice co-infections can override the protective effects of vaccination against other pathogens and increase the susceptibility of fish hosts to other infections, resulting in increased mortality [234,235,236,237].

Recently, numerous studies have been published on the development of vaccines against the salmon sea louse *Lepeophtheirus salmonis*. Even though potential vaccine antigen candidates have been suggested for over 30 years [238,239,240], there is still a bloom in articles concerning the assessment of antigens in laboratory trial settings. In 2018, Swain et al. validated a peptide derived from the salmon lice ribosomal protein (P0) as an interesting antigen candidate against *L. salmonis*, especially when fused with promiscuous T-cell epitopes (TCEs) to boost its efficacy [241]. This approach was shown to be effective in a wide array of fish species, including Tilapia, African catfish, and the Atlantic salmon [242]. In 2020, the same group reported the efficacy of a fused peptide vaccine containing P0 and TCEs, with results supporting an efficacy rate of approximately 56% when administered via IP injection. A relative percentage of protection in the range of ~21% against the adult life stage of the parasite was also reported [243]. Several other salmon lice proteins have been evaluated as vaccine antigens, with the potassium chloride, amino acid transporter (P33) and the Toll-like receptor 6 (P30) proving the most promising [244]. The former candidate was recently reported to provide an adequate and dose-dependent protective effect in Atlantic Salmon against *L. salmonis* when administered via IP injection. A negative correlation was established between P33-specific antibodies in fish plasma and the adult sea lice count, hinting that the intrinsic hematophagous nature of the ectoparasite can potentially be utilized as a strategy for the delivery of salmon-specific antibodies against lice gut proteins [245].

Other attempts have been focused on different sea lice species, such as *Caligus rogercresseyi* and *Caligus elongatus*. While the latter has not gained any traction in terms of experimental vaccination trials as of late, the former has attracted the interest of a research group that published an extensive study exploring three different lice vaccines against the early-stage infestation of *C. rogercresseyi* in Atlantic salmon [246]. In this study, three groups of fish were vaccinated with three different vaccine prototypes containing the recombinant proteins peritrophin, cathepsin, and a mixture of both. The results show a reduction in the early-stage sea lice load, ranging from 25% to 44% in the prototypes containing peritrophin and cathepsin, respectively, when compared to the control group. Similarly, the prototype containing the mixture of both recombinant proteins showed a 52% reduction in the sea lice load. Furthermore, a transcriptomic RNA-Seq analysis was also conducted, showing a prototype-specific modulation of the transcriptome. Collectively, these results underscore the recent progress in parasite vaccine development and the importance of modern and cutting edge -omic tools in assisting this endeavor. Different transcriptional activities can alter the fate of early host–parasite interactions, therefore providing new knowledge on sea lice control in the industry of aquaculture.

#### 4.5.2. Advancing Ciliate Vaccines

Apart from sea lice, however, the aquaculture industry faces challenges from other notable parasites, including ciliates. These parasitic organisms have garnered attention, as researchers aim to develop effective vaccines against them in hopes of alleviating aquaculture from diseases like the marine White Spot Disease (WSD). Traditionally, ciliate immobilization agents (i-antigens) have been identified as promising vaccine antigen candidates due to their ability to elicit cellular immune responses. As the name suggests, these immune responses are materialized through the production of antibodies that immobilize the cilia of the parasites. Specific i-antigens have been successfully identified in two major ciliate parasites in aquaculture, *Ichthyophthirius multifiliis* and Cryptocaryon irritans. DNA and recombinant i-antigen vaccines against the two ciliates have been shown to provide partial protection in relatively recent studies, though the level of protection does not seem to be higher than past attempts utilizing live and killed ciliate theronts [247,248,249].

The use of conventional inactivated vaccines against ciliates is also present in recent studies, as Zhou et al. recently showcased that adding the inactivated form of the ciliate parasite Chilodonella uncinata can boost the efficiency of an inactivated vaccine against I. multifiliis when administered via IP injection in Koi carp fish (Cyprinus carpio) [250]. The efficacy of DNA vaccines encoding I. multifiliis i-antigens was recently shown to be dose-dependent, as higher vaccine doses confer higher levels of antibody titers, the higher upregulation of immune-related genes, and significantly higher survival rates in Channel catfish (Ictalurus punctatus) [251]. The search for potential ciliate antigens appears to continue, as recent attempts are employing cutting-edge, next-generation sequencing transcriptomic analyses to uncover parasite proteases as promising vaccine antigen candidates against cryptocaryoniasis [252]. In a rather recent study conducted by the same research group, a DNA vaccine encoding an infection-related cysteine protease from C. irritans was reported to provide partial protection against parasite infection in Japanese flounder (Paralicthys olivaceus) [253]. Control attempts utilizing this method of vaccination, however, still appear to be in their nascent stages in terms of efficacy.

#### 4.5.3. Innovations in Endoparasite Vaccinology

Vaccination endeavors against endoparasites so far appear to be restricted to three significant parasitic pathogens: the myxozoans *Myxobolus koi* and *Tetracapsuloides bryosalmonae* and the ciliate *Uronema marinum*. Whole and crude spore proteins from *M. koi* have been utilized as both feed immunostimulants and subunit vaccine antigens in goldfish (*Cyprinus caprio*) and have improved survival rates in infected fish [254,255]. In 2019, a DNA vaccination trial study using three different *T. bryosalmonae* antigens administered individually and in combination was published [256]. Partial protection and a reduced endoparasite load were observed in some groups, and a novel micro-exon gene (*Tb*-MEG1) was characterized as a promising vaccine antigen against proliferative kidney disease (PKD) in rainbow trout. Additionally, in 2022, an innovative study was published, where 136 immunogenic proteins of *T. bryosalmonae* were identified by utilizing an in vivo induced antigen assay using infected fish sera to screen a cDNA phage expression library [257].

For *U. marinum*, one of the causative agents of scuticociliatosis, a PLGA-encapsulated vaccine was shown to significantly reduce mortality in kelp grouper (*Epinephelus bruneus*), showcasing that immunoprophylactic nano-formulations can be employed for parasitic diseases too [258]. In addition, a study dealing with the transcriptomic analysis of the gene expression in immune pathways in the spleen of the pufferfish *Takifugu rubripes* after vaccination with an inactivated scuticociliate vaccine was just published in 2024. This study reported approximately 278 differentially expressed genes upon vaccination, yielding valuable insights into gene expression patterns, regulatory mechanisms, and molecular pathways regarding scuticociliate infection [259]. Thus, a sound base can be set for the future treatment and control of endoparasites in the industry of aquaculture, not only nationally but globally, through the establishment of databases for numerous different aquatic species and parasitic pathogens.

## 5. Conclusions

Vaccination in aquaculture stands as a pivotal cornerstone for safeguarding the health of farmed aquatic organisms, representing the most effective preventive measure against infectious diseases. The evolution of aquaculture productivity throughout the years exhibits a direct correlation with the advancement and the utilization of vaccines, which underscores their paramount significance, establishing them as an indispensable component of aquaculture history. Numerous studies substantiate the crucial role of vaccines in bolstering the health and resilience of aquatic species, thereby fortifying the foundations of vaccination in boosting the sustainability of aquaculture in the modern era. This review managed to identify over 80 commercially available aquaculture vaccines licensed for use worldwide, adding a pivotal milestone to a list that is expected to keep expanding in the years to come.

The recent convergence of scientific and technological advancements has led to a substantial bloom in vaccine development capabilities during recent years. Notably, this profound increase stems from understanding various aspects of immunology that were previously unexplored, particularly within the context of aquatic animals. In addition, contemporary breakthroughs in biotechnology and computational biology have provided valuable tools and methodologies that are now essential for the development of new vaccines. This synergy has not only expedited the manufacturing process but has also elevated the precision and efficacy of vaccine production in the industry of aquaculture.

Despite a delayed onset, recent advancements in the manufacturing of aquaculture vaccines are now beginning to take root, driven by the integration of modern and innovative approaches. Current trends highlight a strategic emphasis on enhancing antigen uptake within fish, particularly through advances made in understanding and eliciting mucosal immune responses. This includes not only the exploration of novel delivery routes, such as oral or immersion administration, but also the application of cutting-edge techniques based on nanotechnological applications and modern bioengineering for efficient antigen transfer and high immunization potency. These emerging strategies signify a pivotal shift towards leveraging modern approaches to fortifying the immunization protocols in aquaculture, thereby ensuring enhanced disease prevention and sustainable industry growth in the years to come.

By increasing knowledge on aquatic animal immunology and host–pathogen interactions, the process of aquaculture vaccine development is poised to advance significantly in the future. The contribution of scientific research globally is bound to contribute equally to the quest for sustainable aquatic disease prevention, establishing guidelines pertaining to the multiple interactions between variables that are crucial for vaccine efficacy. Such factors include antigen composition, the route of administration, the type of vaccine technology used, the species and the developmental stage of the host, and the nature of the pathogen in question. By leveraging this approach, and in combination with studies on emerging aquatic pathogens, a structured framework of standards can be established to orient future research endeavors towards increased specificity in vaccine design, thus streamlining the development of aquaculture vaccines tailored to address specific pathogens of interest in the industry.

Overall, the journey of aquaculture vaccine development embodies the dynamic interplay between scientific exploration, technological innovation, and logistical practicality, especially within the context of industry demands. From the foundational milestones in aquaculture vaccine history to the current era of cutting-edge biotechnology and -omic approaches, the narrative reflects a resolute pursuit of solutions to mitigate disease risks and fortify aquatic animal health. The imperative remains to reinforce resilience capacity in the face of evolving pathogens and environmental pressures, ensuring not only the prosperity and sustainability of the sector but also the conservation of wild aquatic ecosystems. Through strategic investments in collaborative research efforts, policies, and education, a direct course can be charted towards a future where aquaculture vaccines can serve as guardians of both profit and biodiversity, fostering an important legacy for the future generations to come.

## Figures and Tables

**Table 3:** A summary and an evaluation of the recent progress on the upcoming aquaculture vaccine technologies collectively discussed in Section 4.

Alternative–Upcoming Vaccine Technologies		
	Advantages	Disadvantages
mRNA Vaccines	High immunogenicity Low manufacturing cost Existing SAV platform Safety	Regulatory restrictions Novelty In need of further research and validation for different species and pathogens
Vector Vaccines	Increased antigen precision Vaccine-delivery capabilities High safety Versatile applications Already implemented	GMO legislation issues Cost effectiveness
Synthetic Peptides—Epitope Vaccines	Maximally immunogenic AI conjunction opportunity Reverse vaccinology approach	Very novel Minimally explored in aquatic species (limited databases)
**New Adjuvants**		
Fish Cytokines	Tailored immune responses Increased vaccination efficacy	Limited knowledge Novelty for aquaculture
Nanoparticle Formulations	Mucosal vaccine delivery Increased vaccination efficacy High versatility Bypassing traditional adjuvant side effects	Relative novelty Cost-related issues Laborious manufacturing
**Oral Vaccination Innovations**		
Polymer Encapsulation	Antigen-integrity protection Non-toxic, mucoadhesive Oral vaccination improvement Relatively inexpensive Nanopolymer options Potential adjuvant effects	Transition to industrial use will require time and effort Effectiveness questions Cost-related questions
Bioencapsulation	Antigen-integrity protection Nutritional benefits Immunomodulatory effects Lifting of size limitations Fry vaccination	Efficacy in question Relative novelty In need of more research Validation for more species
Plant-based Vaccines	Efficient antigen expression Post-translational modifications Edible vaccine opportunities Conjunction with microalgae Bioencapsulation approaches	Very novel in aquaculture In need of more research
**Aquaculture Vaccines for Multicellular Pathogens**		
Sea Lice Vaccine Development	Most well researched Plenty of antigens established Protection for a vastly economically important species (Atlantic Salmon)	Still under development Different sea lice species might need further research
Aquatic Ciliate Vaccines	Establishment of i-antigens DNA vaccination attempts Potential to protect from WSD	Very early stages as of now
Endoparasite Vaccine Progress	DNA vaccination attempts Encapsulation attempts Identification of antigens Transcriptomic insights	Partial protection so far Complexity in development Need for extensive research Limited scope of such vaccines

## Data Availability

Not applicable.
